# Terpenoids from the Roots of *Stellera chamaejasme* (L.) and Their Bioactivities

**DOI:** 10.3390/molecules28237726

**Published:** 2023-11-23

**Authors:** Juan Wu, Zhujun Ye, Caicen Liao, Rongtao Li, Xuanqin Chen

**Affiliations:** 1 Faculty of Life Science and Technology, Kunming University of Science and Technology, Kunming 650500, China; 20213118002@stu.kust.edu.cn (J.W.); 201911806171@stu.kust.edu.cn (Z.Y.); 20202118032@stu.kust.edu.cn (C.L.); 2 Key Laboratory of New Drugs (Traditional Chinese Medicine) for Respiratory Viral Diseases of Yunnan Province, Kunming 650500, China

**Keywords:** *Stellera chamaejasme* L, stellerterpenoids A–C, ECD calculation, NO inhibition

## Abstract

An undescribed diterpene, stellerterpenoid A (**1**), and two undescribed sesquiterpenoids, stellerterpenoids B and C (**2**–**3**), together with six known compounds, prostratin (**4**) stelleraguaianone B (**5**), chamaejasnoid A (**6**), auranticanol L (**7**), wikstronone C (**8**), and oleodaphnone (**9**), were isolated from the roots of *Stellera chamaejasme* L. Their structures were elucidated by extensive spectroscopic data (1D, 2D NMR, IR, UV, and HR-ESI-MS). The absolute configuration of **1**–**3** was elucidated based on ECD calculation. Among them, stellerterpenoid A was a rare 13, 14-seco nortigliane diterpenoid and stellerterpenoid B was a guaiacane-type sesquiterpenoid with an unusual 1, 2-diketone moiety. The known stelleraguaianone B (**5**) exhibited moderate activity for suppressing NO production in lipopolysaccharide (LPS)-treated RAW 264.7 macrophages cells with an IC_50_ value of 24.76 ± 0.4 μM. None of the compounds showed anti-influenza virus or anti-tumor activity in vitro.

## 1. Introduction

The genus *Stellera* is comprised of 10-12 species, two of which are distributed in China (*Stellera chamaejasme* Linn. and *Stellera formosana* Hayata ex Li) [1]. *S. chamaejasme* L, also known as ‘Lang Du’, is a toxic plant that is widely distributed in north and southwest China [2,3]. The issue of *S. chamaejasme* spreading is increasingly serious as it consumes soil moisture and nutrients [4]. The coverage of forage plants and grassland yield were greatly decreased due to the uncontrolled spread of *S*. chamaejasme, which competes with other plants for nutrient space and weakens the normal growth of herbage [5]. The roots of *S. chamaejasme* can be used to extract industrial alcohol and make paper due to its high content of wolfsbane fiber [6]. Although the whole plant of *S. chamaejasme* L is poisonous, it has certain medicinal values. *S. chamaejasme* was widely used in traditional Chinese medicine (TCM) for the effects of clearing heat, detoxification, detumescence, reducing inflammation, stopping ulcers, and removing saprophytic muscle [7,8,9,10,11]. Many studies have been conducted on its chemical composition and pharmacological activities in recent decades. A series of compounds have been reported, including highly oxidized daphnane-type diterpenes [12,13], guaiane-type sesquiterpenoids [14,15,16,17,18], unusual C-3/C-3″-biflavanones [19,20,21,22], lignans [14], and coumarins [20]. Some compounds from *S. chamaejasme* have displayed many different and interesting biological activities in modern pharmacological research. For example, gnidimacrin, a 1-alkyldaphnane-type diterpene, was found to have strong anti-cancer activity with a broad anti-cancer spectrum [23]. Daphnane diterpenes, stelleralides D-J, exhibited more potent anti-HIV activity (EC_50_ = 0.73~0.98 nM) than zidovudine (positive controls, EC_50_ = 32 nM) [24]. Moreover, chamechromone can significantly inhibit the expression of proinflammatory cytokines in RAW 264.7 cells [25]. According to the above literature, the diterpenoids and sesquiterpenoids are possibly the active components in this plant owing to the anti-inflammatory, anti-viral, and anti-tumor activities.

**Figure 1:**
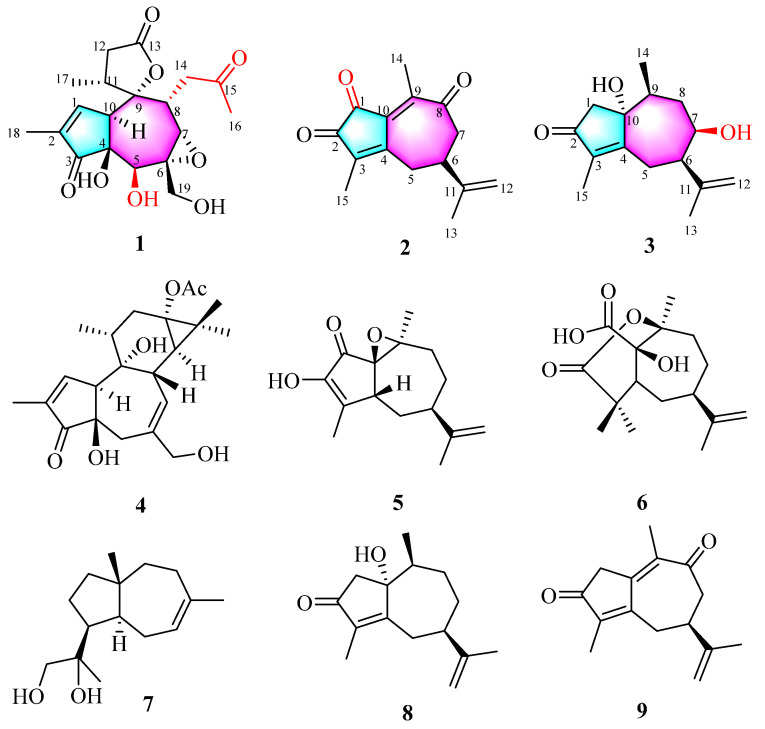
Structures of compounds **1**–**9** isolated from *Stellera chamaejasme* L.

In order to identify the natural compounds with anti-inflammatory, anti-viral, and anti-tumor activities from *S. chamaejasme*, the methanol extract of the roots were phytochemically investigated in our continuous research work. As a result, three new terpenoids, including stellerterpenoid A (**1**) with a rare 13, 14-*seco* nortigliane skeleton, stellerterpenoid B (**2**) with an unusual 1, 2-diketone moiety, and a guaiacane sesquiterpenoid stellerterpenoid C (**3**), together with six known terpenoids (**4**–**9**), were identified from the ethyl acetate extract (Figure 1). In addition, the anti-inflammatory, anti-influenza virus, and anti-tumor activities of the nine compounds were tested. Herein, the isolation, identification, structural characterization, and biological assessment of these compounds are reported.

## 2. Results and Discussion

### 2.1. Structural Elucidation of Three New Compounds (***1***, ***2***, and ***3***)

**Figure 2:**
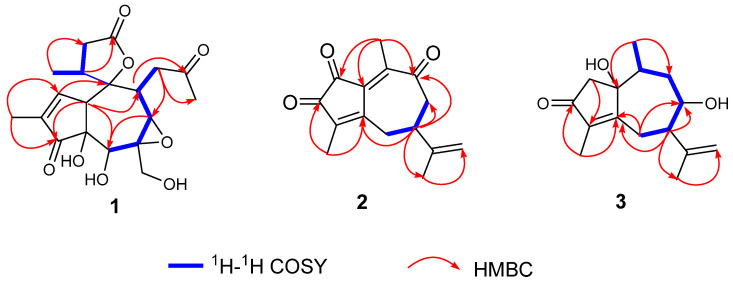
^1^ H–^1^ H COSY and key HMBC correlations of compounds **1**–**3**.

Stellerterpenoid A (**1**), obtained as a yellow oil, had a molecular formula of C_19_H_24_O_8_ deduced from the quasi-molecular ion peak at *m*/*z* 403.1344 [M + Na]^+^ (calcd 403.1363) in the HR-ESI-MS (Figure S7), with eight degrees of unsaturation. The ^1^H NMR spectrum (Table 1) of **1** displayed the presence of three methyls at *δ*_H_ 2.19 (s, H-16), 1.78 (s, H-18), and 1.18 (d, *J* = 7.0 Hz, H-17), an oxymethines at *δ*_H_ 3.85 (s, H-19), a suite of methines including an olefinic proton at *δ*_H_ 7.48 (d, *J* = 1.8 Hz, H-1), and four methines at *δ*_H_ 4.31 (s, H-5), 3.57 (ddd, *J* = 10.6, 5.4, 2.4 Hz, H-8), 3.24 (d, *J* = 6.0 Hz, H-7), and 2.74 (m, H-11). Its ^13^C NMR and DEPT spectra (Table 2) revealed 19 carbon signals, including three methyls [*δ*_c_ 30.2 (C-16), 18.0 (C-17), and 10.0 (C-18)], three methylenes [*δ*_c_ 63.6 (C-19), 42.6 (C-14), and 38.9 (C-12)], six methines [*δ*_c_ 158.3 (C-1), 75.9 (C-5), 58.8 (C-7), 51.0 (C-10), and 40.3 (C-8)], and seven quaternary carbons consisting of two carbonyl groups [*δ*_C_ 209.8 (C-3) and 209.2 (C-15)], one ester carbonyl group at *δ*_C_ 177.9 (C-13), one olefinic group at *δ*_C_ 137.7 (C-2), and three oxygenated ones at *δ*_C_ 91.8 (C-9), 76.2 (C-4), and 66.0 (C-6). The 5/7 carbocyclic ring system was determined via HMBC analysis. The HMBC correlations from H-18 to C-1, C-2, and C-3 indicated that C-3 and C-1 were connected through C-2 (Figure 2). The HMBC correlations from H-5 to C-3, C-4, and C-10 suggested that C-3 was linked to C-10 and C-5 via C-4 (Figure 2). The HMBC correlations of H-10 with C-8 and C-9 proved that C-9 was combined with C-8. In addition, C-5 was linked to C-7 and C-6 due to the HMBC correlations from H_2_-19 to C-5, C-6, and C-7 (Figure 2). 

The ^1^H and ^13^C-NMR spectral data of **1** were similar to those of crotonianoid A [26] except for the occurrence of the signals due to a methylene group (*δ*_C_ 42.6, C-14) and a ketone carbonyl (*δ*_C_ 209.2, C-15) in **1** instead of the *∆*^14,15^ double bond (*δ*_C_ 123.0, 136.5) in crotonianoid A. The phenomena suggested that the *∆*^14,15^ double bond in crotonianoid A was oxidized to the methylene and ketone carbonyl in **1**. In addition, the methylene of C-5 [*δ*_C_ 38.8, *δ*_H_ 2.41 (d, *J* = 18.6 Hz), 2.66 (d, *J* =18.6 Hz)] in crotonianoid A was replaced by one oxy-methine [*δ*_C_ 75.9, *δ*_H_ 4.31 (s)], and the double bond was replaced with one epoxy structure at C-6 and C-7. The C-9 and C-13 are similar to crotonianoid A, and this was demonstrated by signal offset via an oxygen atom generating a five-membered lactone ring [26]. Furthermore, the HMBC cross-peaks from H-8 (δ_H_ 3.57) to C-14 (*δ*_C_ 42.6) and C-15 (*δ*_C_ 209.2) and from H-16 (*δ*_H_ 2.19) to C-14 (*δ*_C_ 42.6) and C-15 (*δ*_C_ 209.2) supported the location of the acetone unit at C-8 (Figure 2). 

**Figure 3:**
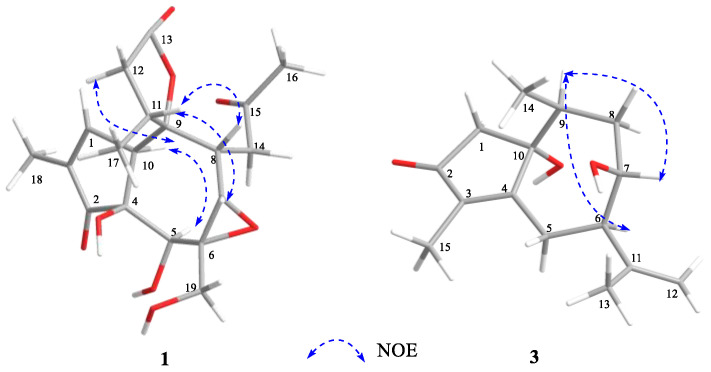
Key NOESY correlations of compounds **1** and **3**.

**Figure 4:**
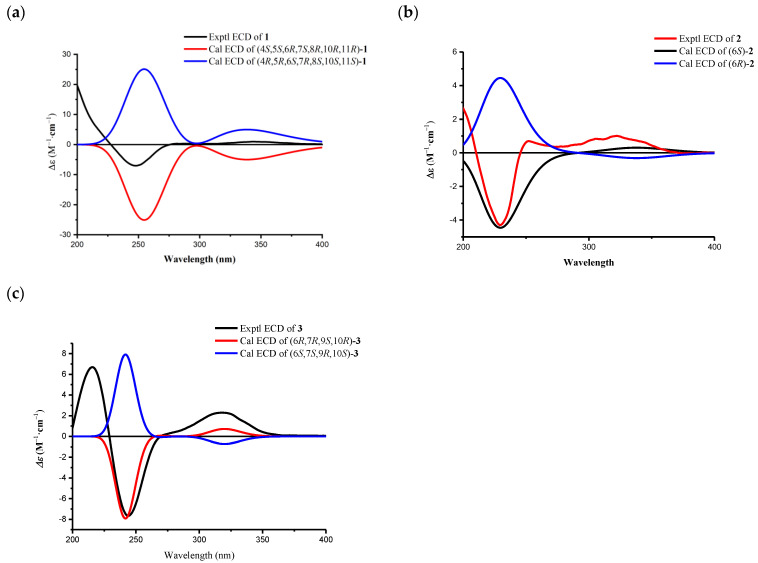
Calculated and experimental ECD spectra of compounds **1**–**3**.

The relative configuration of **1** was elucidated by the analysis of NOESY spectrum. The NOESY correlations of H-7/H-11, H-8/H-11, and H-8/H-12*β* (Figure 3) allowed the H-7, H-8, H-11, and C-9-C-11 to be assigned the *β*-orientation, while the NOESY cross-peaks of H-10/H-5, H-5/H-17, and H-12*α*/H-17 indicated that H-5, H-10, and Me-17 were *α*-oriented. The negative cotton effect at 250 nm in the experimental ECD curve of **1** assigned its absolute configuration as 4*S*, 5*S*, 6*R*, 7*S*, 8*R*, 10*R*, and 11*R* (Figure 4a). Therefore, the structure of **1** was fully elucidated to be a rare 13, 14-seco nortigliane diterpenoid and named stellerterpenoid A. To the best our knowledge, it is the second report of 13, 14-*seco* nortigliane diterpenoid other than crotonianoid A.

**Scheme 1:**
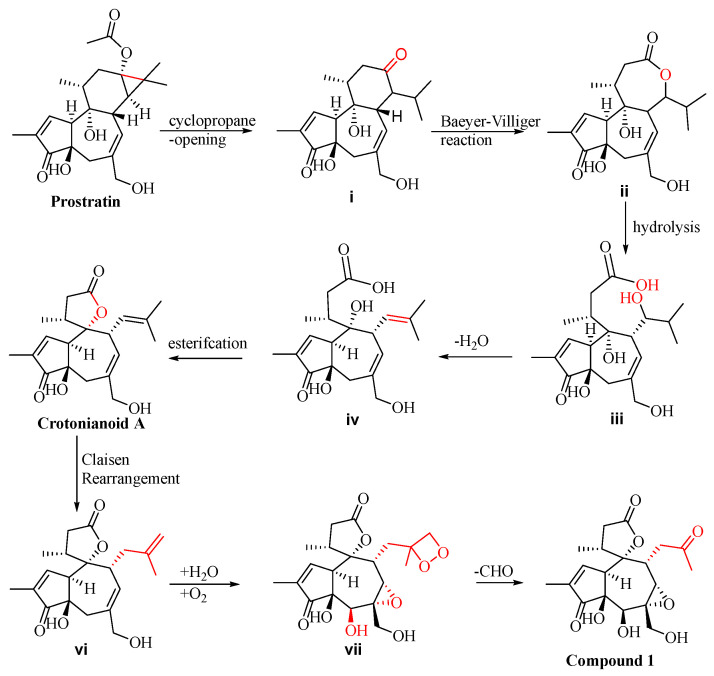
Proposed Biosynthetic Pathway for **1**.

The plausible biosynthetic pathways of **1** were proposed in Scheme 1. Crotonianoid A originated from prostratin through the intermediate products i, ii, iii, and iv [26]. Furthermore, the C-14 and C-15 double bond in crotonianoid A were rearranged to C-15 and C-16 by claisen rearrangement to obtain intermediate vi, which were oxidized to obtain vii, and HCHO was then removed to obtain compound **1**.

Stellerterpenoid B (**2**) was obtained as a yellow oil with a molecular formula of C_15_H_16_O_3_ according to its HR-ESI-MS at *m*/*z* 267.0981 [M + Na]^+^ (calcd 267.0992) (Figure S14), suggesting eight degrees of unsaturation. The ^1^H NMR spectrum (Table 1) of **2** exhibited one pair of double bonds at *δ*_H_ 4.81 (s, H-12b) and 4.80 (s, H-12a) and three methyl peaks at *δ*_H_ 1.95 (s, H-14), 1.85 (s, H-15), and 1.80 (s, H-13). The ^13^C NMR spectrum (Table 2), in combination with the DEPT spectrum, showed 15 carbon signals, including three methyls in the high magnetic field at *δ*_c_ 20.4 (C-13), 17.3(C-14), and 8.8 (C-15), an olefinic carbon at *δ*_c_ 111.3 (C-12), two methylenes at *δ*_c_ 50.6 (C-7) and 36.3 (C-5), one methine at *δ*_c_ 40.3 (C-6), and eight quaternary carbons at *δ*_c_ 204.3 (C-2), 202.6 (C-1 and C-8), 165.1 (C-4), 148.9 (C-11), 146.1 (C-3), 145.9 (C-10), and 132.6 (C-9). The NMR data indicated that **2** was a guaiacane sesquiterpene.

The NMR spectral data of **2** were very similar to those of oleodaphnone [27] except for the replacement of one ketone carbonyl in **2** by one methylene in oleodaphnone. This observation suggested that one methylene in oleodaphnone was oxygenated into one ketone carbonyl in **2**. The HMBC correlation from H-14 (*δ*_H_ 1.95) to C-1 (*δ*_C_ 202.6) verified that the ketone was located at C-1 (Figure 2). The experimental ECD spectrum of **2** demonstrated the negative cotton effect at 230 nm and the positive cotton effect at 327 nm, which agreed well with the calculated ECD spectrum of (6*S*)-**2** (Figure 4b). Therefore, the structure of **2** was elucidated to be a guaiacane sesquiterpene with an unusual 1,2-diketone moiety and named stellerterpenoid B.

**Scheme 2:**
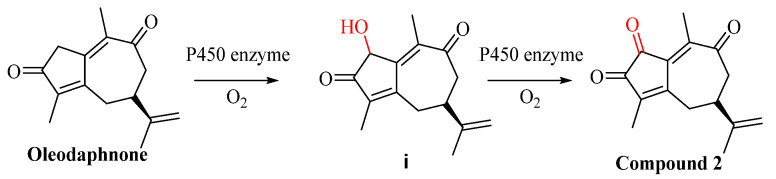
Proposed Biosynthetic Pathway for **2**.

Since a 1,2-diketone fragment is rare in natural compounds, the biosynthetic pathway of **2** was proposed based on oleodaphnone (Scheme 2). The C-1 of oleodaphnone is oxidized by P450 enzyme to form hydroxyl intermediate **i**, which is further oxidized to form **2** [28].

Stellerterpenoid C (**3**) was obtained as a colorless oil. Its HR-ESI-MS exhibited a quasi-molecular ion peak at *m*/*z* 273.1454 [M + Na]^+^ (calcd 273.1461) (Figure S21), suggesting a molecular formula of C_15_H_22_O_3_ with five degrees of unsaturation. The ^1^H NMR spectroscopic data (Table 1) show the presence of three methyls at *δ*_H_ 1.80 (s, H-13), 1.64 (s, H-15), and 0.85 (d, *J* = 7.2 Hz, H-14), six methylenes at *δ*_H_ 4.79 (s, H-12a), 4.76 (s, H-12b), 2.57 (d, *J* = 18.2 Hz, H-1a), 2.34 (d, *J* = 18.2 Hz, H-1b), 2.20 (ddd, *J* = 13.8, 10.0, 3.5 Hz, H-8), and 1.75 (ddd, *J* = 13.8, 5.4, 3.5 Hz, H-8), and three methines at *δ*_H_ 3.69 (m, H-7), 3.00 (m, H-6), and 2.29 (m, H-9). The ^13^C NMR spectrum showed 15 signals which were ascribed to three methyls at *δ*_C_ 19.9 (C-13), 15.6 (C-14), and 7.9 (C-15), four methylenes at *δ*_C_ 112.8 (C-12), 50.3 (C-1), 32.2 (C-5), and 40.8 (C-8), three methylates at *δ*_C_ 70.5 (C-7), 51.3 (C-6), and 37.9 (C-9), and five quaternary carbons at *δ*_C_ 208.3 (C-2), 138.9 (C-3), 173.8 (C-4), 82.2 (C-10), and 149.0 (C-11) (Table 2). 

The NMR spectra of **3** were extremely similar to those of known wikstronone C [29], except that the signals of one methylene were replaced by one oxy-methine in **3**. The above-mentioned NMR spectral feature suggested that one methylene was oxygenated into one oxy-methine in **3**. The HMBC correlations from H-7 (*δ*_H_ 3.69) to C-8 (*δ*_C_ 40.8) and C-6 (*δ*_C_ 51.3), together with the ^1^H-^1^H COSY spin system of H-6/H-7/H-8/H-9, indicated that a hydroxy was connected with C-7 in **3** (Figure 2). In the NOESY spectrum, CH_3_-14 (*δ*_H_ 0.85) correlated with H-1*β* (*δ*_H_ 2.57), suggesting that CH_3_-14 is *β*-oriented. The NOESY correlations of H-7/H-9 and H-6/H-9 suggested the *α*-orientation of H-6 and H-7 (Figure 3). The 10-OH is defined as the *β*-configuration because the chemical shift of C-10 (*δ*_C_ 82.2) is similar to that (*δ*_C_ 83.2) of wikstronone C. The positive cotton effect at 216 nm and the negative cotton effect at 244 nm in experimental ECD spectrum of **3** (Figure 4c) assigned its stereochemistry as 6*R*, 7*R*, 9*S*, and 10*R*. Hence, the structure of **3** was characterized and named stellerterpenoid C.

The six known compounds were identified by 1D NMR spectral analysis and compared with the reported data in the references to be prostratin (**4**) [30], stelleraguaianone B (**5**) [14], chamaejasnoid A (**6**) [31], auranticanol L (**7**) [32], wikstronone C (**8**) [29], and oleodaphnone (**9**) [27].

### 2.2. Biological Studies

Compounds **1**–**9** were evaluated for their inhibition on nitric oxide (NO) production, stimulated by lipopolysaccharide (LPS) in RAW 264.7 macrophages. The results showed that compound **5** displayed moderate activity for inhibiting NO production (IC_50_ = 24.76 ± 0.4 μM) and had no cytotoxic effect on RAW 264.7 macrophages (CC_50_ > 50 μM). N^G^—monomethyl-l-arginine, monoacetate salt (L-NMMA) was used as the positive control (IC_50_ = 12.30 ± 3.4 μM) (Table 3). 

The inhibitory rate of HepG2, A549, and HeLa cell growth of compounds **1**–**9** was measured by the MTT assay. Unfortunately, none of these nine compounds were active (IC_50_ > 50 μM). 

Furthermore, compounds **1**–**9** were also tested for anti-influenza virus activity against A/Puerto Rico/8/1934 (H1N1) virus. However, none of the compounds showed inhibitory activity (EC_50_ > 50 μM). 

## 3. Materials and Methods

### 3.1. General Experimental Procedures

Optical rotations were recorded on a Jasco P-1020 polarimeter (JASCO Corporation, Tokyo, Japan). CD spectra were obtained on a Jasco J715 spectrometer polarimeter. UV spectra were measured using a UV-8000 spectrophotometer (Shanghai Metash instruments Co., Ltd., Shanghai, China). IR spectra were recorded on a Bio-Rad FTS-135 spectrometer. (Bio-rad Company, Hercules, CA, USA). NMR data were determined by Bruker Avance III HD-600 (Bruker BioSpin Group, Rheinstetten, Germany). ESI-MS and HR-ESI-MS were recorded on an APIQ star-Pulsar spectrometer (Applied Biosystems Corporation, Ontario, Canada). The rotary evaporator is an n-1300V-W type (Tokyo Physicochemical Co., Ltd., Kyoto, Japan). Semi-preparative HPLC was carried out on an Agilent 1260 system (ZORABAX SB-C18, 4.6 mm × 250 mm, 1 mL/min; 9.4 mm × 250 mm, 3 mL/min) (Agilent Technology Co., Ltd., Santa Clara, CA, USA) and NP7005-10C (CC: Nucifera C8M, 4.6 mm × 250 mm, 1 mL/min; 20 × 250 mm,10 mL/min) (Hanbon Sci. and Tech, Jiangsu, China). Methanol and acetonitrile (pure reagents) were purchased from Merck Company (Merck, Darmstadt, Germany). Sephadex LH-20 (25-100 μm) was purchased from Amersham Biosciences AB (Uppsala, Sweden). Thin layer silica gel plates (GF254) were purchased from Qingdao Marine Chemical Company (Qingdao, China) and spots were visualized by heating silica gel plates sprayed with 5% H_2_SO_4_-EtOH.

### 3.2. Plant, Virus Strain, and Cancer Cell Materials

The fresh roots of *S*. *chamaejasme* L were collected in Dali city, Yunnan Province, People’s Republic of China, on 12 August 2021, and identified by Dr Zhijun Zhang, Kunming University of Science and Technology. A voucher specimen (KUMST20211007) has been deposited at the Key Laboratory of Phytochemistry, Kunming University of Science and Technology. The RAW 264.7 was purchased from Conservation Genetics CAS Kunming Cell Bank (KCB200603YJ). The A/PR/8/34 (HIN1) virus strain was donated by Professor Yang Zifeng from Guangzhou Medical University, China. The A549 (human lung cancer cells), HepG2 (liver cancer cells), and HeLa (cervical cancer cells) were donated by Nanjing KGI Biotechnology Company (Nanjing, China).

### 3.3. Extraction and Isolation

The fresh roots of *S*. *chamaejasme* L were air-dried. Dried roots of *Stellera chamaejasme* (11.0 kg) were crushed and extracted with 70% acetone/H_2_O three times (3 × 50 L) at room temperature to give a crude extract (10.1 kg) under reduced pressure distillation. The extract was mixed with water (15.0 L), followed by successive partitioning with petroleum ether (3 × 15 L) and EtOAc (3 × 15 L). The EtOAc extract (3.35 kg) was separated by silica gel column chromatography (25 × 200 cm) using a gradient of petroleum ether/EtOAc (5:1-1:1, *v*/*v*) and CHCl_3_/MeOH (3:1–1:1, *v*/*v*) as the eluents to give eight fractions (Fr. A~H).

Fr. C (45.75 g) was separated by using medium pressure column (MeOH, *v*/*v*, 30:70~90:10) and then using silica gel column chromatography (10 × 160 cm) with a gradient solvent petroleum ether/EtOAc (5:1–1:1, *v*/*v*) to obtain six fractions (Fr. C1~C6). 

Fr. C3 (3.3 g) was repeatedly separated by silica gel column chromatography (5 × 70 cm, CH_2_Cl_2_/MeOH, 10:1–1:1, *v*/*v*) to obtain four fractions (Fr. C3-1~C3-4). Fr. C3-1 (198.4 mg) was separated using silica gel column chromatography (1 × 70 cm, CH_2_Cl_2_/MeOH, 10:1-1:1, *v*/*v*) to obtain Fr. C3-1-1~Fr. C3-1-4. Fr. C3-1-1 (45.7 mg) was purified by Sephadex LH-20 (2.5 × 120 cm, CH_2_Cl_2_/MeOH, 1:1, *v*/*v*) to yield **2** (8.3 mg). Fr. C3-1-2 (53.1 mg) was purified by semi-preparative HPLC (MeOH/H_2_O, 65:35, *v*/*v*) to obtain **5** (13.3 mg). Fr. C3-1-3 (34.0 mg) was separated by Sephadex LH-20 (2.5 × 120 cm, MeOH) and semi- preparative HPLC (MeOH/H_2_O, 61:39, *v*/*v*) to obtain **6** (3.7 mg) and **7** (6.2 mg). Fr. C3-1-4 (45.8 mg) was further segmented by a silica gel chromatography column washed with petroleum ether-isopropanol (3:1) to yield **8** (12.4 mg). Fr. C3-2 (1.2 g) was applied to a silica gel (3 × 60 cm) column chromatography washed with petroleum ether-acetone (15:1–1:1, *v*/*v*) to provide fractions of C3-2-1~C3-2-6. Fr. C3-2-2 (550.0 mg) was applied to silica gel (2 × 70 cm, CH_2_Cl_2_/isopropanol, 8:1-1:1, *v*/*v*) column chromatography to divide it into five fractions (Fr. C3-2-2-1~Fr. C3-2-2-5). Fr. C3-2-2-2 (63.7 mg) was further separated by semi-preparative HPLC (MeOH/H_2_O, 57:43, *v*/*v*) to obtain **3** (3.7 mg). Fr. C3-2-2-4 (62.5 mg) was separated by Sephadex LH-20 (1.5 × 120 cm, MeOH) and semi-preparative HPLC (MeOH/H_2_O, 55:45, *v*/*v*) to obtain **9** (3.8 mg). 

Fr. C4 (7.5 g) was subjected to silica gel (7 × 160 cm) using CHCl_3_/MeOH (20:1–1:1, *v*/*v*) as the mobile phase to give six fractions (Fr. C4-1~C4-6). Fr. C4-2 (840 mg) was separated by silica gel column chromatography (2 × 60 cm, CHCl_3_/MeOH, 15:1-1:1, *v*/*v*) to obtain fractions of C4-2-1~C4-2-3. Fr. C4-2-2 (355.8 mg) was segmented by silica gel column chromatography eluted with dichloromethane-methanol (15:1) to obtain three subfractions (Fr. C4-2-2-1~ C4-2-2-3). Fr. C4-2-2-1 (34.5 mg) was purified by Sephadex LH-20 eluted with CHCl_3_-MeOH (1:1, *v*/*v*) to yield **1** (39.3 mg). Fr. C4-2-2-2 (283.5 mg) was used with silica gel (CHCl_3_-MeOH, 15:1, *v*/*v*, 2 × 70 cm) column chromatography to yield **4** (105.3 mg). 

### 3.4. Details of New Compounds

#### 3.4.1. Stellerterpenoid A (**1**)

Yellow oil; [*α*${]}_{D}^{24}$−37.1 (c 0.24, MeOH); UV (MeOH) *λ*_max_ (log ε) 244 (1.88) nm; IR (KBr) *ν*_max_ 3449, 2918, 2920, 1769, 1703, 1631, 1424, 1361, 1292, 1220, 1164, 1089, 1040, 966, 930, 834, 800, 724, 676, 585, 530, and 418 cm^−1^; for ^1^H NMR (CD_3_OD, 600 MHz) and ^13^C NMR (CD_3_OD, 150 MHz) data, see Table 1 and Table 2; HR-ESI-MS *m*/*z* 403.1344 [M + Na]^+^ (calcd 403.1363).

#### 3.4.2. Stellerterpenoid B (**2**)

Yellow oil; [*α*${]}_{D}^{24}$−11.0 (c 0.05, MeOH); UV (MeOH) *λ*_max_ (log ε) 207 (1.09) nm, 230 (1.01) nm, and 301 (1.59) nm; IR (KBr) *ν*_max_ 3444, 2919, 2851, 1702, 1647, 1444, 1383, 1309, 1262, 1179, 1092, 896, and 802 cm^−1^; for ^1^H NMR (CD_3_OD, 600 MHz) and ^13^C NMR (CD_3_OD, 150 MHz) data, see Table 1 and Table 2; HR-ESI-MS *m*/*z* 267.0981 [M + Na]^+^ (calcd 267.0992).

#### 3.4.3. Stellerterpenoid C (**3**)

Colorless oil; [*α*${]}_{D}^{24}$+91.8 (c 0.10, MeOH); UV (MeOH) *λ*_max_ (log ε) 239 (1.56) nm; IR (KBr) *ν*_max_ 3458, 3070, 2972, 2928, 2852, 1688, 1629, 1454, 1382, 1319, 1230, 1188, 1164, 1110, 1050, 978, 878, 807, 729, 655, 552, and 463 cm^−1^; for ^1^H NMR (CD_3_OD, 600 MHz) and ^13^C NMR (CD_3_OD, 150 MHz) data, see Table 1 and Table 2; HR-ESI-MS *m*/*z* 273.1454 [M + Na]^+^ (calcd 273.1461).

### 3.5. ECD Calculations for ***1***, ***2***, and ***3***

Based on the conformation determined from ROESY spectra and Chem 3D modeling, the low-energy conformers of model compounds (4*S*,5*S*,6*R*,7*S*,8*R*,10*R*,11*R*)-**1**, (6*S*)-**2**, and (6*R*,7*R*,9*S*,10*R*)-**3** were generated within a 10 kcal/mol energy window under MMFF94S via the software CONFLEX [33]. The selected conformers of each model compound with the highest distribution (fuef63, fuef4, and fuef72-1) of conformers for compounds **1**–**3**, respectively, were further optimized by the density functional theory method at the B3LYP/6-31G (d) level. The ECD calculations were performed using level TD-DFT-B3LYP/6-311G (+, 2d, p) of the theory on optimized geometries through the CPCM model (in MeOH) [34]. The calculated ECD curves were generated using SpecDis 1.71 [35] and all of the above calculations were carried out with the Gaussian 16 package of programs. All ECD curves were weighted by Boltzmann distribution after UV correction.

### 3.6. Determination of NO Production

The murine macrophage cell line RAW 264.7 was cultured in Dulbecco’s modified Eagle’s medium (DMEM) supplemented with 10% fetal bovine serum (FBS) and 1% penicillin (10,000 U/mL)-streptomycin (10,000 μg/mL) at 37 °C in a humidified incubator containing 5% CO_2_. The RAW 264.7 (8 × 10^4^/well) cells were left to acclimate for 24 h before any treatments and were seeded onto 96-well plates and pretreated by compounds **1**–**9** (3.125–50 µM) 1 h prior to treatment with 1 μg/mL LPS. Afterward, co-stimulation for 24 h at 37 °C was carried out in an incubator under 5% CO_2_. Then, Griess reagents I and II (100 µL) were mixed with cell culture medium (70 µL) and incubated at room temperature for 10 min with horizontal shaking, after which the absorbance was measured at 540 nm using a microplate reader (Thermo Fisher Scientific, Waltham, MA, USA). N(G)-monomethyl-Larginine, monoacetate salt (L-NMMA) and DMSO were used as positive and negative controls, respectively [36].

### 3.7. Anti-Influenza Virus Assay

Influenza strain A/Puerto Rico/8/1934 (H1N1) was used in this study. MDCK cells (1 × 10^4^/well) were inoculated in 96-well plates and cultured for 24 h before viral infection. The infection medium was MEM (0.1 mM non-essential amino acid, 1% FBS, 1 mM sodium pyruvate, 1 µg/mL trypsin (Sigma-Aldrich, Beijing, China), and 100 U/mL penicillin/streptomycin). The cells were treated with various concentrations (from 3.125 μM to 50 μM) of tested compounds **1**–**9** and detected by Promega (Madison, WI, USA) CellTiter-Glo^®^ reagent following the protocol provided by the supplier. The emitted luminescence (RLU) was quantified with a Promega Victor III plate reader. Oseltamivir was used as the positive control [37].

### 3.8. Anti-Tumor Assay

HepG2, A549, and HeLa cell lines were used for cytotoxic activity. Cells were cultured in DMEM medium at 37 °C in 5% CO_2_. The medium was composed of 10% FBS, 1% HEPES, 1% mixed penicillin (10,000 U/mL), and streptomycin (10,000 μg/mL) fluid, as well as 10% fetal bovine serum. The cells (1 × 10^5^ per well) were seeded on 96-well microplates and allowed to adhere for 12 h before drug addition. Each cell line was treated with compounds **1**–**9** at different concentrations (3.125–50 μM) in triplicate for 48 h. Spectra-Max M2 (Molecular Devices Inc, San Jose, CA, USA) was employed to record the optical density (*λ* = 490 nm). IC_50_ values were calculated based on the mean OD values measured three times versus the concentration curves of drugs. Adriamycin (10 mM, purity 99%, Solar bio Science and Technology Co., Ltd., Beijing, China) and DMSO were used as the positive and negative control [38].

### 3.9. Statistical Analysis

All biological experiments were repeated at least three times and the results were obtained using Statistical Package for Social Sciences (SPSS Version 21.0) software [36]. 

## 4. Conclusions

In summary, three previously unreported terpenoids including a diterpene (**1**), two guaiacane sesquiterpenoids (**2**–**3**), and six known ones (**4**–**9**) were isolated from the roots of *Stellera chamaejasme*. Their structures were elucidated by extensive spectroscopic data (1D, 2D NMR, IR, UV, and HR-ESI-MS). The absolute configuration of compounds **1**–**3** were established by the experimental and calculated ECD. Compound **5** exhibited moderate NO inhibitory activity in LPS-induced RAW 264.7 macrophages (IC_50_ = 24.76 ± 0.4 μM). None of the compounds showed anti-influenza virus and anti-tumor activity in vitro.

## Figures and Tables

**Table 1:** The ^1^H-NMR data (600 MHz, *δ*_H_, mult, *J* in Hz) of **1**–**3** in CD_3_OD.

No.	1	2	3
1	7.48 d (1.8)		2.57 d (18.2)
			2.34 d (18.2)
2			
3			
4			
5	4.31 s	3.03 d (17.2)	2.69 d (6.7)
		2.89 d (6.5)	
6		2.76 m	3.00 m
7	3.24 d (6.0)	2.94 m	3.69 m
		2.85 m	
8	3.57 ddd (10.6, 5.4, 2.4)		2.20 ddd (13.8, 10.0, 3.5)
			1.75 ddd (13.8, 5.4, 3.5)s
9			2.29 m
10	3.47 d (2.6)		
11	2.74 m		
12	3.09 dd (18.1, 8.1)	4.81 s	4.79 s
	2.27 dd (18.1, 4.4)	4.80 s	4.76 s
13		1.80 s	1.80 s
14	2.83 dd (18.1, 10.6)	1.95 s	0.85 d (7.2)
	2.63 dd (18.1, 2.6)		
15		1.85 s	1.64 s
16	2.19 s		
17	1.18 d (7.0)		
18	1.78 s		
19	3.85 s		

**Table 2:** The ^13^C-NMR data (150 MHz, *δ*_C_) of **1**–**3** in CD_3_OD.

No.	1	2	3
1	158.3	202.6	50.3
2	137.7	204.3	208.3
3	209.8	146.1	138.9
4	76.2	165.1	173.8
5	75.9	36.3	32.2
6	66.0	40.3	51.3
7	58.8	50.6	70.5
8	40.3	202.6	40.8
9	91.8	132.6	37.9
10	51.0	145.9	82.2
11	39.2	148.9	149.0
12	38.9	111.3	112.8
13	177.9	20.4	19.9
14	42.6	17.3	15.6
15	209.2	8.8	7.9
16	30.2		
17	18.0		
18	10.0		
19	63.6		

**Table 3:** Inhibitory effects of compounds **1**–**9** on LPS-induced NO production in RAW 264.7 macrophages.

Compds	^a^ IC_50_ (μM)	^b^ CC_50_ (μM)	Compds	IC_50_ (μM)	CC_50_ (μM)
**1**	>50	^c^ NT	**6**	>50	NT
**2**	>50	NT	**7**	>50	NT
**3**	>50	NT	**8**	>50	NT
**4**	>50	NT	**9**	>50	NT
**5**	24.76 ± 0.4	>50	**^d^ L-NMMA**	12.30 ± 3.4	>50

^a^ IC_50_: 50% inhibitory concentration. ^b^ CC_50_: 50% cytotoxic concentration. ^c^ NT represents compounds which were not tested due to insufficient amounts. ^d^ L-NMMA (N^G^-Monomethyl-L-arginine, monoacetate salt) was used as the positive control.

## Data Availability

The data presented in this study are available in the Supplementary Materials.

## Supplementary Materials

The following supporting information can be downloaded at: https://www.mdpi.com/article/10.3390/molecules28237726/s1. Figure S1. ^1^H-NMR spectrum (600 MHz, CD_3_OD) of compound **1.** Figure S2. ^13^C-NMR spectrum (150 MHz, CD_3_OD) of compound **1.** Figure S3. HSQC spectrum of compound **1.** Figure S4. HMBC spectrum of compound **1.** Figure S5. COSY spectrum of compound **1.** Figure S6. NOESY spectrum of compound **1.** Figure S7. HR-ESI-MS spectrum of compound **1.** Figure S8. ^1^H-NMR spectrum (600 MHz, CD_3_OD) of compound **2.** Figure S9. ^13^C-NMR spectrum (150 MHz, CD_3_OD) of compound **2.** Figure S10. HSQC spectrum of compound **2.** Figure S11. HMBC spectrum of compound **2.** Figure S12. COSY spectrum of compound **2.** Figure S13. NOESY spectrum of compound **2.** Figure S14. HR-ESI-MS spectrum of compound **2**. Figure S15. ^1^H-NMR spectrum (600 MHz, CD_3_OD) of compound **3.** Figure S16. ^13^C-NMR spectrum (150 MHz, CD_3_OD) of compound **3.** Figure S17. HSQC spectrum of compound **3.** Figure S18. HMBC spectrum of compound **3.** Figure S19. COSY spectrum of compound **3.** Figure S20. NOESY spectrum of compound **3.** Figure S21. HR-ESI-MS spectrum of compound **3.** Table S1. The energy distribution and boltzmann weights of each configuration of compound (4S, 5S, 6R,7S, 8R, 10R, 11R)-1. Table S2. The energy distribution and boltzmann weights of each configuration of compound (6*S*)-**2.** Table S3. The energy distribution and boltzmann weights of each configuration of compound (6*R*, 7*R*, 9*S*, 10*R*)-**3.** Table S4. Standard orientation of 1a. Table S5. Standard orientation of 1b. Table S6. Standard orientation of 1c. Table S7. Standard orientation of 1d. Table S8. Standard orientation of 1e. Table S9. Standard orientation of 1f. Table S10. Standard orientation of 1g. Table S11. Standard orientation of 1h. Table S12. Standard orientation of 2a. Table S13. Standard orientation of 2b. Table S14. Standard orientation of 2c. Table S15. Standard orientation of 2d. Table S16. Standard orientation of 2e. Table S17. Standard orientation of 2f. Table S18. Standard orientation of 2g. Table S19. Standard orientation of 2h. Table S20. Standard orientation of 3a. Table S21. Standard orientation of 3b. Table S22. Standard orientation of 3c. Table S23. Standard orientation of 3d. Table S24. Standard orientation of 3e. Table S25. Standard orientation of 3f. Table S26. Standard orientation of 3g. Table S27. Standard orientation of 3h. Table S28. Standard orientation of 3i. Table S29. Standard orientation of 3j. Table S30. Standard orientation of 3k.

## References

[1] Song Q., Li S.F., Cheng Z.Y., Song S.J., Huang X.X. (2023). Chemical constituents from *Stellera chamaejasme* L. and chemotaxonomic significance. Biochem. Syst. Ecol..

[2] Liu X.N., Yang Q., Zhang G.L., Li Y.J., Chen Y., Weng X.G., Wang Y.J., Wang Y.W., Zhu X.X. (2014). Anti-tumor pharmacological evaluation of extracts from *Stellera chamaejasme* L. based on hollow fiber assay. BMC Complem. Altern. M..

[3] Zhang C., Zhou S.S., Feng L.Y., Zhang D.Y., Lin N.M., Zhang L.H., Pan J.P., Wang J.B., Li J. (2013). In vitro anti-cancer activity of chamaejasmenin B and neochamaejasmin C isolated from the root of *Stellera chamaejasme* L. Acta Pharmacol. Sin..

[4] Guo L.Z., Li J.H., He W., Liu L., Huang D., Wang K. (2019). High nutrient uptake efficiency and high water use efficiency facilitate the spread of *Stellera chamaejasme* L. in degraded grasslands. BMC Ecol..

[5] Jiang Y.J., Li Q.H., Mao W.Q., Tang W.T., White J.F.J., Li H.Y. (2022). Endophytic bacterial community of *Stellera chamaejasme* L. and its role in improving host plants competitiveness in grasslands. Environ. Microbiol..

[6] Li J., Shen Q., Bao C.H., Chen L.T., Li X.R. (2014). A new dicoumarinyl ether from the roots of *Stellera chamaejasme* L. Molecules.

[7] Zhang N., He J., Xia C.Y., Lian W.W., Yan Y., Ding K., Zhang Y.Y., Xu J.K., Zhang W.K. (2021). Ethnopharmacology, phytochemistry, pharmacology, clinicalapplications and toxicology of the genus *Stellera* Linn.: A review. J. Ethnopharmacol..

[8] Jo B.G., Bong S.K., Jegal J., Kim S.N., Yang M.H. (2020). Antiallergic effects of phenolic compounds isolated from *Stellera chamaejasme* on RBL-2H3 cells. Nat. Prod. Commun..

[9] Li J., Zhao W., Hu J.L., Cao X., Yang J., Li X.R. (2011). A new C-3/C-3′- biflavanone from the roots of *Stellera chamaejasme* L. Molecules.

[10] Wang Z.X., Cheng M.C., Zhang X.Z., Hong Z.L., Gao M.Z., Kan X.X., Li Q., Wang Y.J., Zhu X.X., Xiao H.B. (2014). Cytotoxic biflavones from *Stellera chamaejasme*. Fitoterapia.

[11] Wang K.Y., Wang Z.Y., Wang Z.Q., Xie X.L., Zang L.L., Wang L.J., Che F.Y. (2022). *Stellera chamaejasme* L. extracts in the treatment of glioblastoma cell lines: Biological verification based on a network pharmacology approach. Front. Oncol..

[12] Yoshida M., Feng W.J., Saijo N., Ikekawa T. (1996). Antitumor activity of daphnane-type diterpene gnidimacrin isolated from *Stellera chamaejasme* L. Int. J. Cancer.

[13] Asada Y., Sukemori A., Watanabe T., Malla K.J., Yoshikawa T., Li W., Koike K.Z., Chen C.H., Akiyama T., Qian K.D. (2011). Stelleralides A-C, novel potent anti-HIV daphnane-type diterpenoids from *Stellera chamaejasme* L. Org. Lett..

[14] Jing C.X., Guo J.J., Yang B.J., Fan S.R., Wang Y.T., Chen D.Z., Hao X.J. (2019). Stelleraguaianone B and C, two new sesquiterpenoids from *Stellera chamaejasme* L. Fitoterapia.

[15] Hu F.F., Qi D.D., Xu S., Mao W. (2021). A new 11, 10-guaiane-type sesquiterpenoid from the roots of *Stellera chamaejasme* Linn. J. Chem. Res..

[16] Pan J., Su J.C., Liu Y.H., Deng B., Hu Z.F., Wu J.L., Xia R.F., Chen C., He Q., Chen J.C. (2021). Stelleranoids A-M, guaiane-type sesquiterpenoids based on [5,7] bicyclic system from *Stellera chamaejasme* and their cytotoxic activity. Bioorg. Chem..

[17] Cheng Z.Y., Hou Z.L., Ren J.X., Zhang D.D., Huang X.X., Lin B., Song S.J. (2021). Guaiane-type sesquiterpenoids from the roots of *Stellera chamaejasme* L. and their neuroprotective activities. Phytochemistry.

[18] Cheng Z.Y., Zhang D.D., Ren J.X., Li Y.L., Yao G.D., Song S.J., Huang X.X. (2022). Stellerasespenes A-E: Sesquiterpenoids from *Stellera chamaejasme* and their anti-neuroinflammatory effects. Phytochemistry.

[19] Asada Y., Sukemori A., Watanabe T., Malla K.J., Yoshikawa T., Li W., Kuang X.Z., Koike K., Chen C.H., Akiyama T. (2013). Isolation, structure determination, and anti-HIV evaluation of tigliane-type diterpenes and biflavonoid from *Stellera chamaejasme*. J. Nat. Prod..

[20] Jiang Z.H., Tanaka T., Sakamoto T., Kouno I., Duan J.A., Zhou R.H. (2002). Biflavanones, diterpenes, and coumarins from the roots of *Stellera chamaejasme* L. Chem. Pharm. Bull..

[21] Jo B.G., Park N.J., Jegal J., Choi S., Lee S.W., Jin H., Kim S.N., Yang M.H. (2018). A new flavonoid from *Stellera chamaejasme* L., stechamone, alleviated 2,4-dinitrochlorobenzene-induced atopic dermatitis-like skin lesions in a murine model. Int. Immunopharmacol..

[22] Yan Z.Q., Guo H.R., Yang J.Y., Liu Q., Jin H., Xu R., Cui H.Y., Qin B. (2014). Phytotoxic flavonoids from roots of *Stellera chamaejasme* L. (Thymelaeaceae). Phytochemistry.

[23] Yoshida M., Feng W.J., Nishio K., Takahashi M., Heike Y.J., Saijo N., Wakasugi H., Ikekawa T. (2001). Antitumor action of the PKC activator gnidimacrin through CDK2 inhibition. Int. J. Cancer.

[24] Yan M., Lu Y., Chen C.H., Zhao Y., Lee K.H., Chen D.F. (2015). Stelleralides D-J and anti-HIV daphnane diterpenes from *Stellera chamaejasme*. J. Nat. Prod..

[25] Kim M., Lee H.J., Randy A., Yun J.H., Oh S.R., Nho C.W. (2017). *Stellera chamaejasme* and its constituents induce cutaneous wound healing and anti-inflammatory activities. Sci. Rep..

[26] Hu R., Huang J.L., Yuan F.Y., Wei X., Zou M.F., Tang G.H., Li W., Yin S. (2022). Crotonianoids A-C, three unusual tigliane diterpenoids from the seeds of *Croton tiglium* and their anti-prostate cancer activity. J. Org. Chem..

[27] Hitomi T., Yoshihisa T., Gisho H., Yasuhiro I., Ekrem S., Erdem Y. (1999). Terpenoids and aromatic compounds from *Daphne oleoides* ssp. Oleoides. Phytochemistry.

[28] Salamanca Karande D., Schmid A., Dobslaw D. (2015). Novel cyclohexane monooxygenase from *Acidovorax* sp. CHX100. Appl. Microbiol. Biotechnol..

[29] Liu Z.H., Dong M.Y., Chang H., Han N., Yin J. (2020). Guaiane type of sesquiterpene with NO inhibitory activity from the root of *Wikstroemia indica*. Bioorg. Chem..

[30] Gustafson K.R., Cardellina J.H., McMahon J.B., Gulakowski R.J., Ishitoya J., Szallasi Z., Lewin N.E., Blumberg P.M., Weislow O.S., Beutler R.W. (1992). A nonpromoting phorbol from the Samoan medicinal plant *Homalanthus nutans* inhibits cell killing by HIV-1. J. Med. Chem..

[31] Wu M.B., Shao J.Q., Zhu J.X., Zi J.C. (2022). Chamaejasnoids A-E, a 2,3-*seco*-guaiane sesquiterpenoid with a 5/6/7 bridged ring system and related metabolites from *Stellera chamaejasme* L. Fitoterapia.

[32] Huang S.Z., Zhang X., Ma Q.Y., Zheng Y.T., Dai H.F., Wang Q., Zhou J., Zhao Y.X. (2015). Anti-HIV terpenoids from *Daphne aurantiaca* Diels Stems. RSC Adv..

[33] Fujihara T., Obora Y., Tokunaga M., Sato H., Tsuji Y. (2005). Dendrimer N- heterocyclic carbene complexes with rhodium (I) at the core. Chem. Commun..

[34] Pescitelli G., Bruhn T. (2016). Good computational practice in the assignment of absolute configuration by TDDET calculations of ECD spectra. Chirality.

[35] Bruhn T., Schaumlöffel A., Hemberger Y., Pescitelli G. (2017). SpecDis.

[36] Yang Y.F., Liu T.T., Li G.X., Chen X.Q., Li R.T., Zhang Z.J. (2023). Flavonoids from the roots of *Sophora flavescens* and their potential anti-inflammatory and antiproliferative activities. Molecules.

[37] Jiang N., Quan L.Q., Zhou Y., Cheng Y.Y., Li H.M., Chen X.Q., Li R.T., Liu D. (2022). Exploring the anti-influenza virus activity of novel triptolide derivatives targeting nucleoproteins. Bioorg. Chem..

[38] Khiev P., Kim J.W., Sung S.J., Song H.H., Choung D.H., Chin Y.W., Lee H.K., Oh S.R. (2012). Ingenane-type diterpenes with a modulatory effect on IFN-*γ* production from the roots of *Euphorbia kansui*. Arch Pharm. Res..

