# Supplementary material for: Terpenoids from the Roots of Stellera chamaejasme (L.) and Their Bioactivities
Source: Molecules. 2023 Nov 23;28(23):7726. doi: 10.3390/molecules28237726 (PMC10707970; doi:10.3390/molecules28237726)
Supplement: Supplementary file 1 [file molecules-28-07726-s001.zip › molecules-2682092-supplementary.pdf]

Supplementary Material

Terpenoids from the Roots of *Stellera chamaejasme* (L.) and Their Bioactivities

Juan Wu [1], Zhujun Ye [1], Caicen Liao [1], Rongtao Li [1,2,*] and Xuanqin Chen [1,2,*]

[1] Faculty of Life Science and Technology, Kunming University of Science and Technology, Kunming 650500, China

[2] Key Laboratory of New Drugs (Traditional Chinese Medicine) for Respiratory Viral Diseases of Yunnan Province, Kunming 650500, China

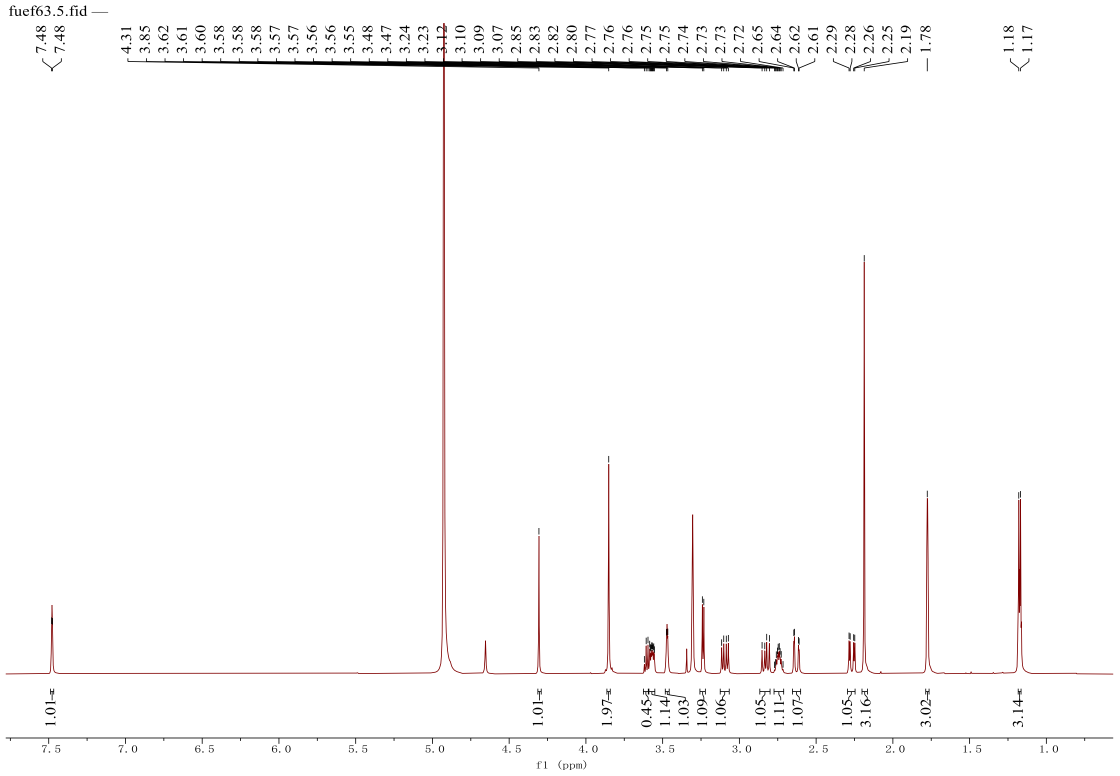

Figure S1. ^{1}H-NMR spectrum (600 MHz, CD_3OD) of compound **1.**

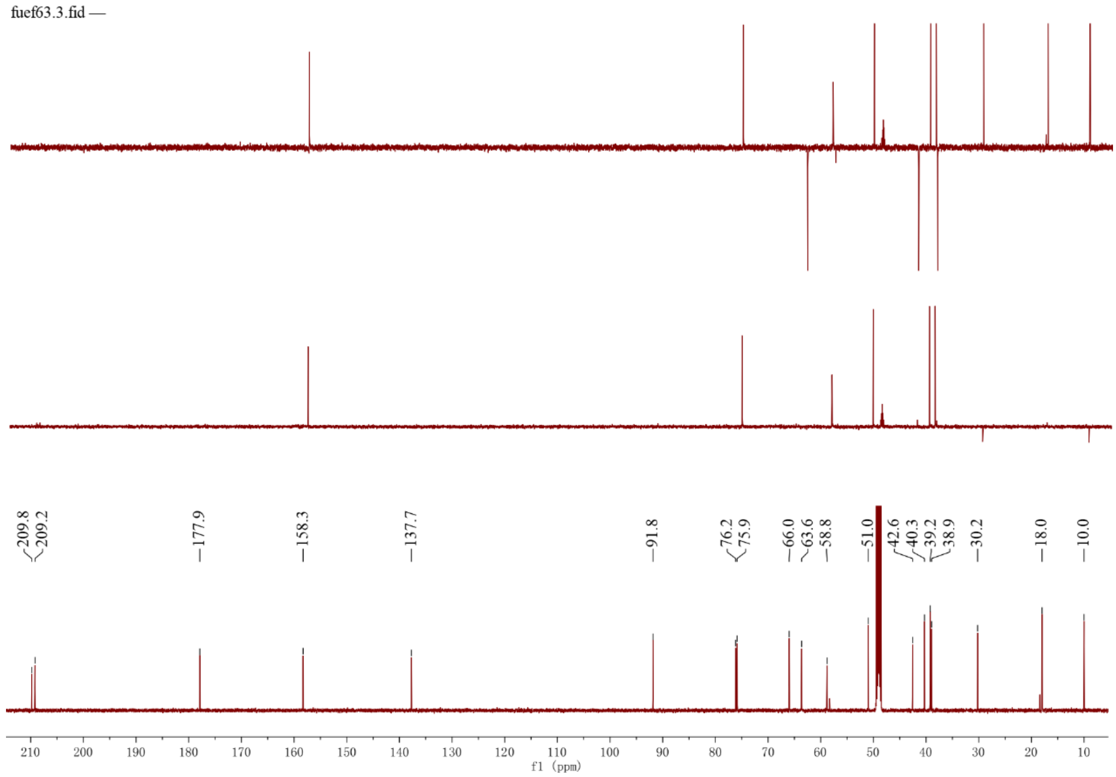

Figure S2. ^{13}C-NMR spectrum (150 MHz, CD_3OD) of compound **1.**

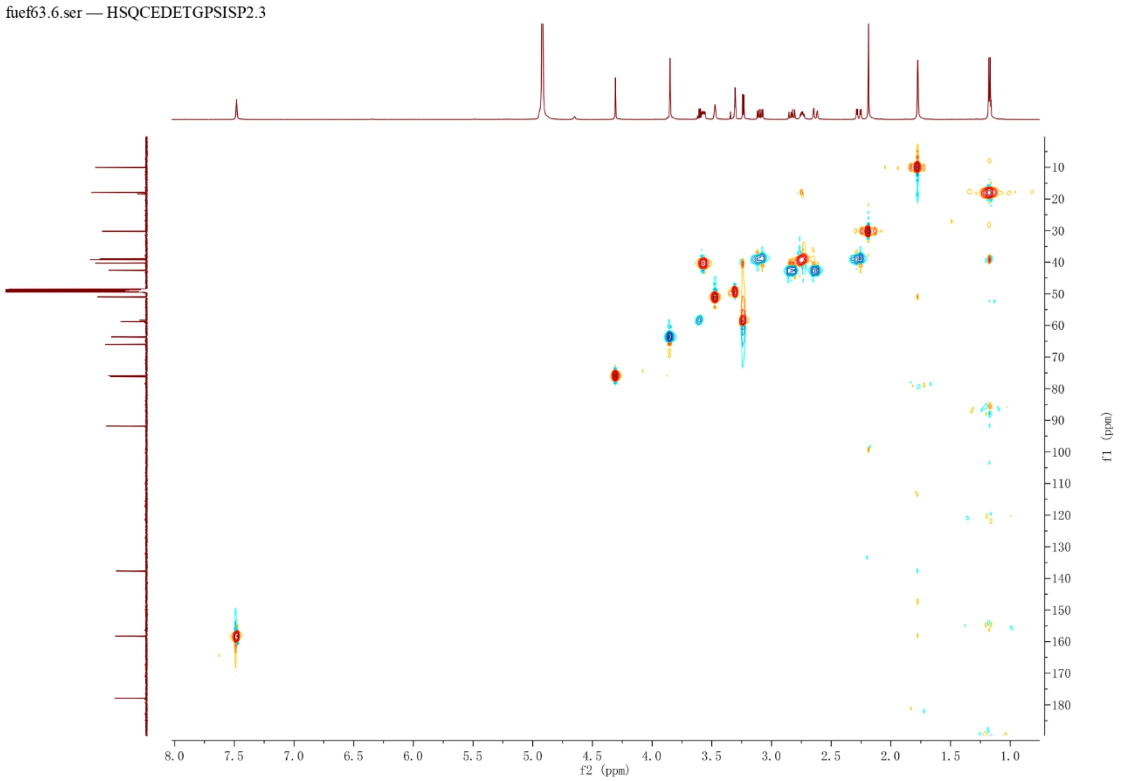

Figure S3. HSQC spectrum of compound **1.**

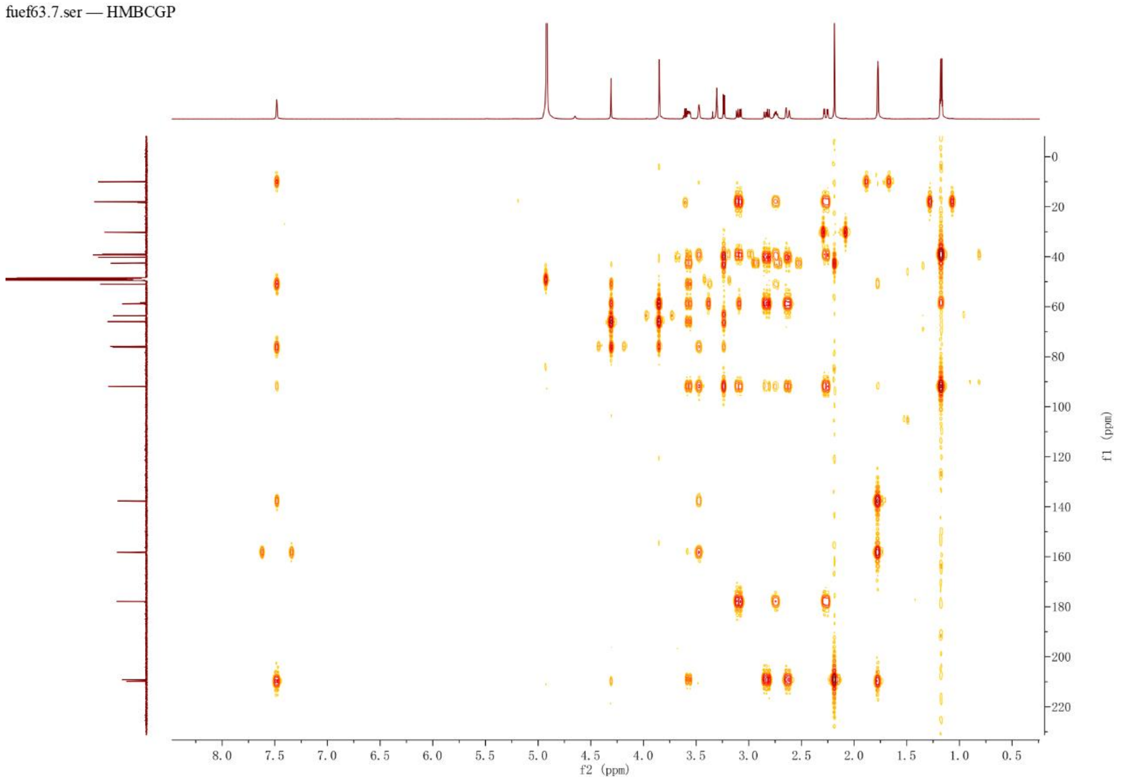

Figure S4. HMBC spectrum of compound **1.**

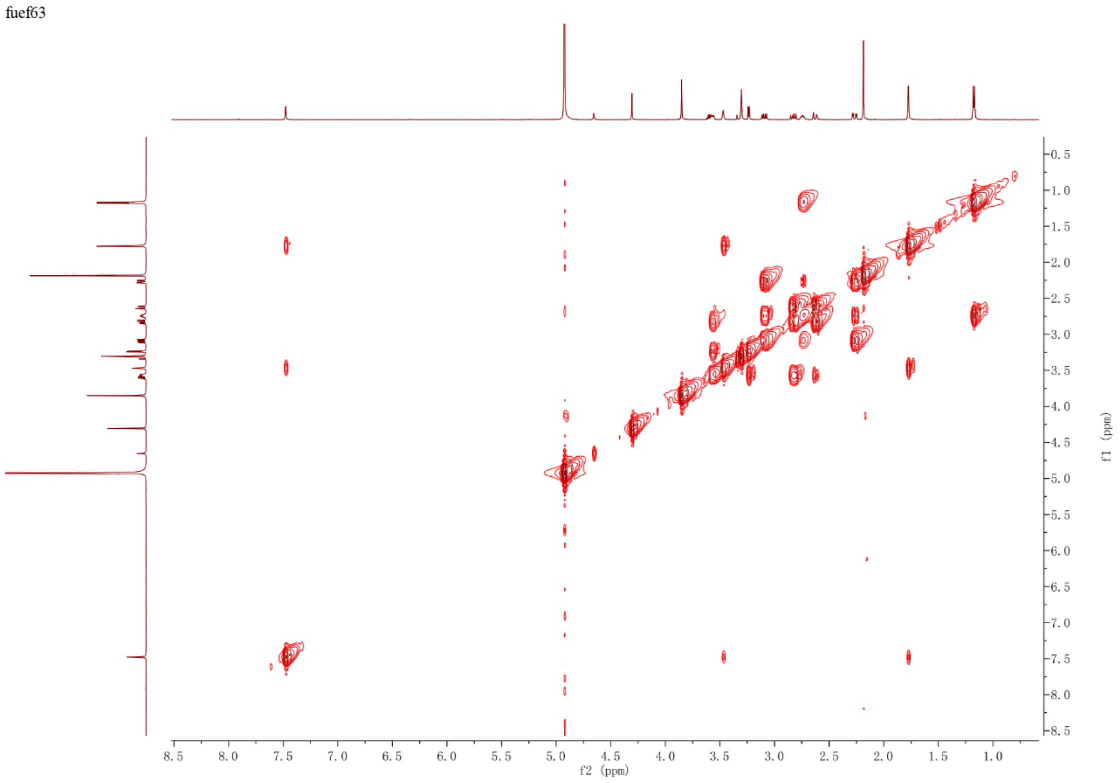

Figure S5. COSY spectrum of compound **1.**

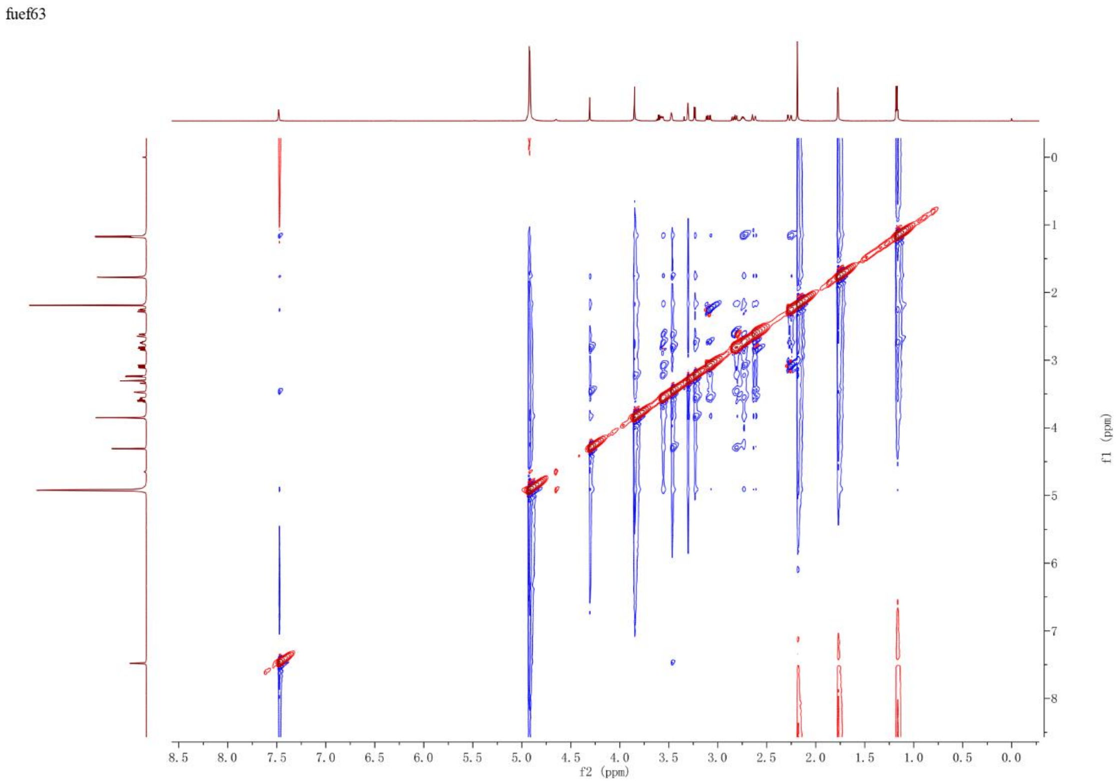

Figure S6. NOESY spectrum of compound **1.**

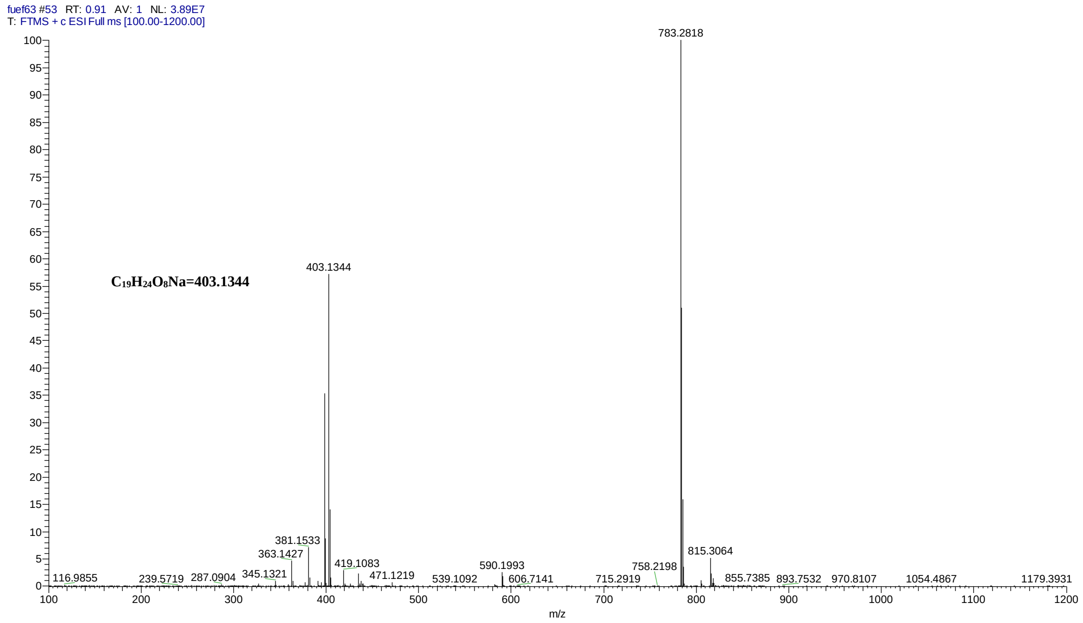

Figure S7. HR-ESI-MS spectrum of compound **1.**

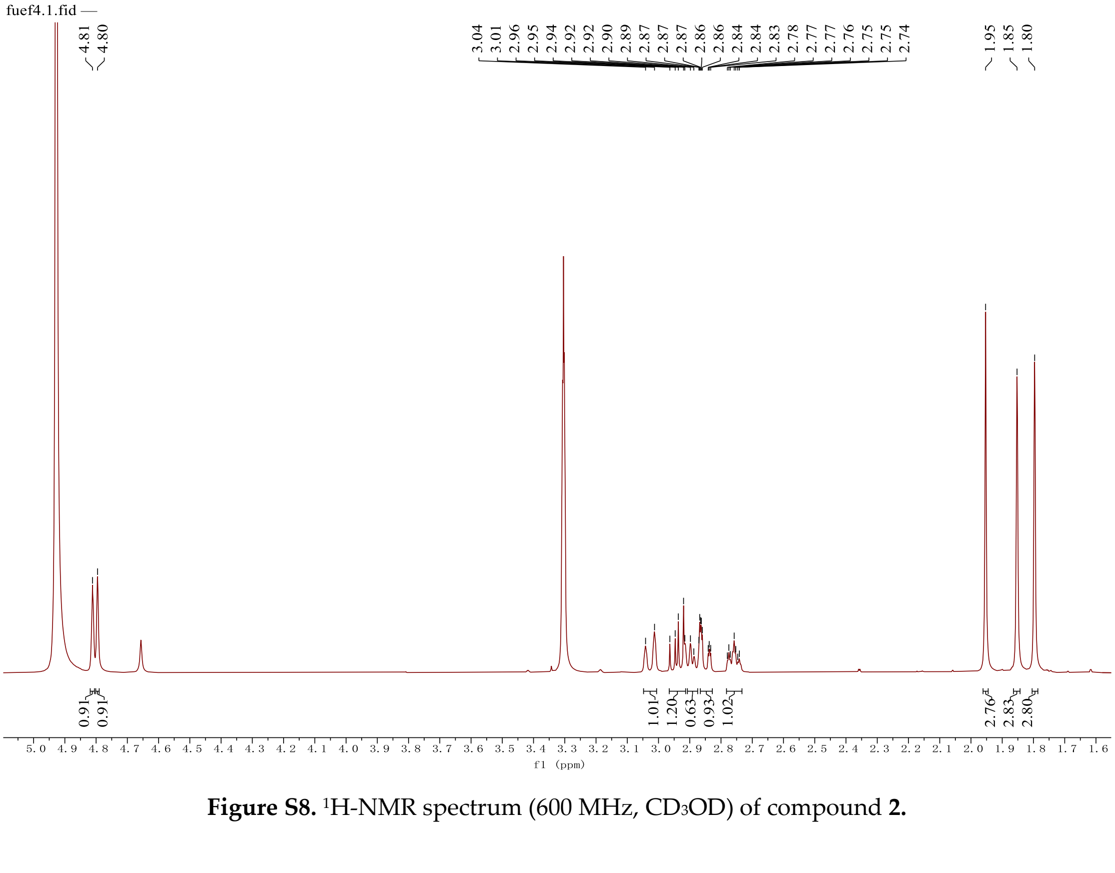

Figure S8. ^{1}H-NMR spectrum (600 MHz, CD_3OD) of compound **2.**

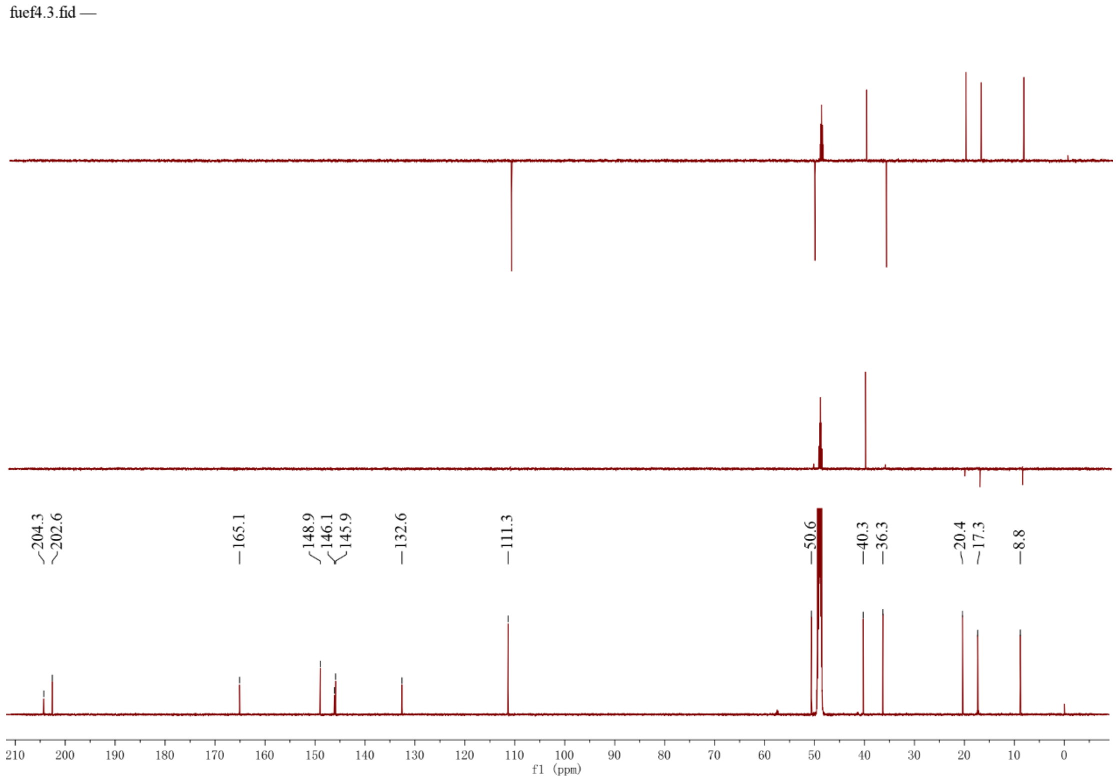

Figure S9. ^{13}C-NMR spectrum (150 MHz, CD_3OD) of compound **2.**

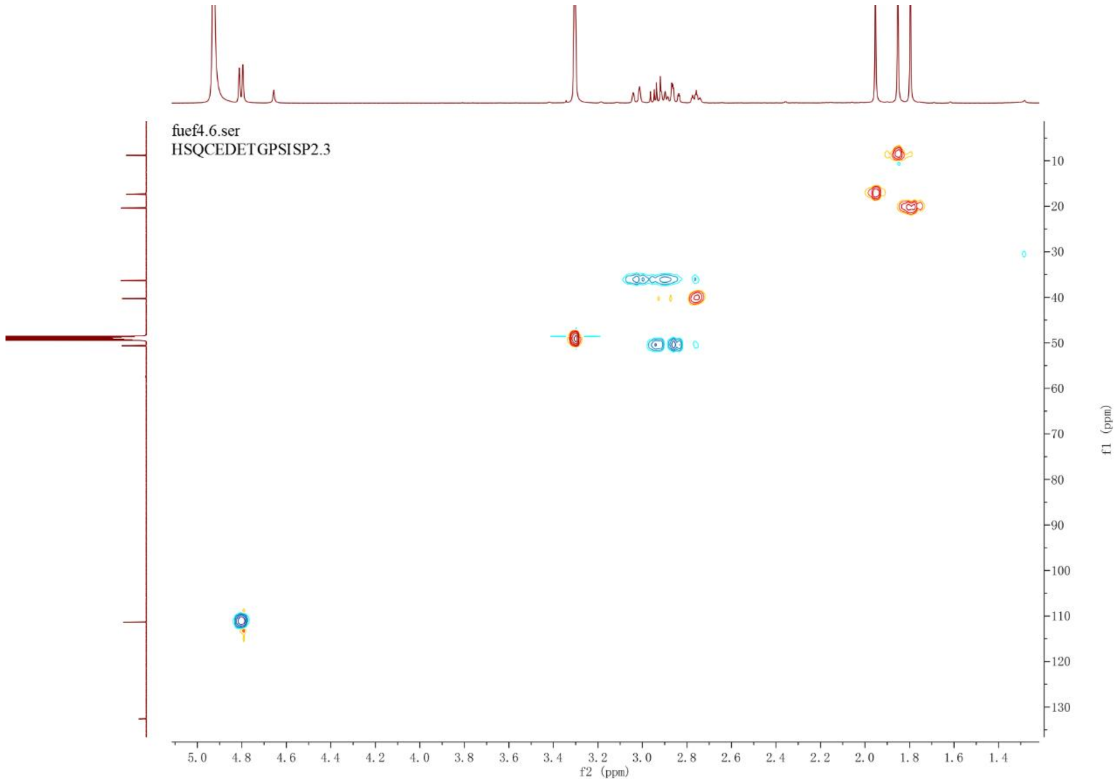

Figure S10. HSQC spectrum of compound **2.**

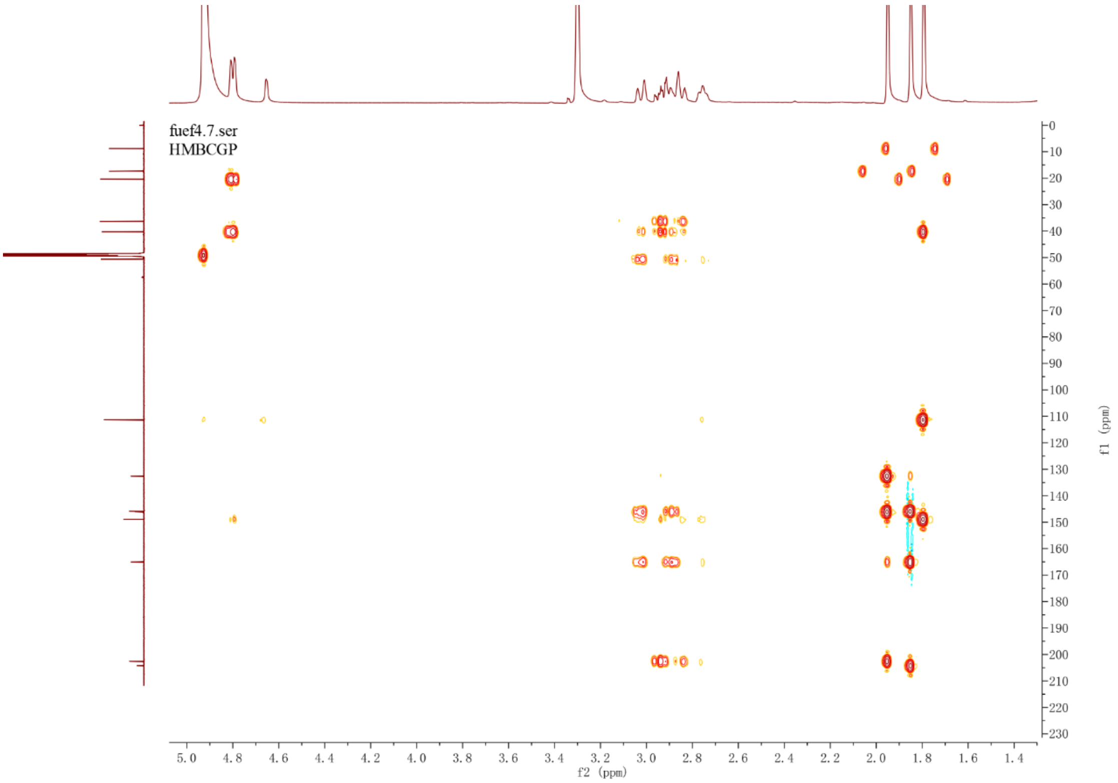

Figure S11. HMBC spectrum of compound **2.**

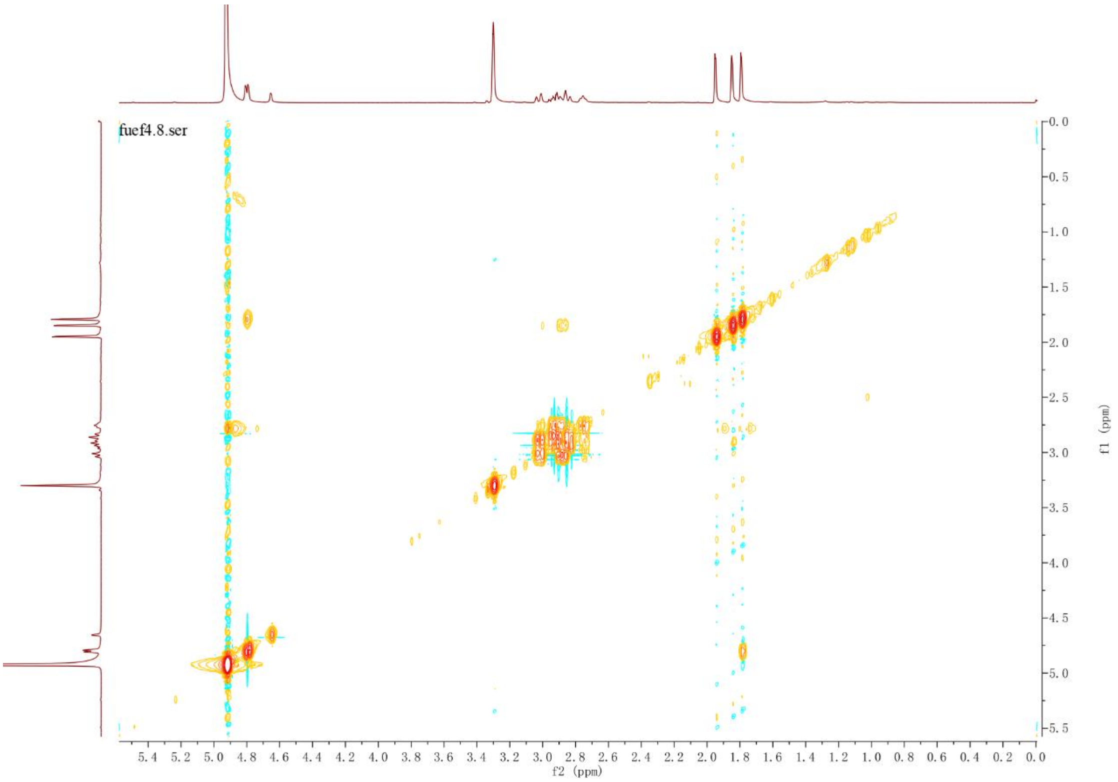

Figure S12. COSY spectrum of compound **2.**

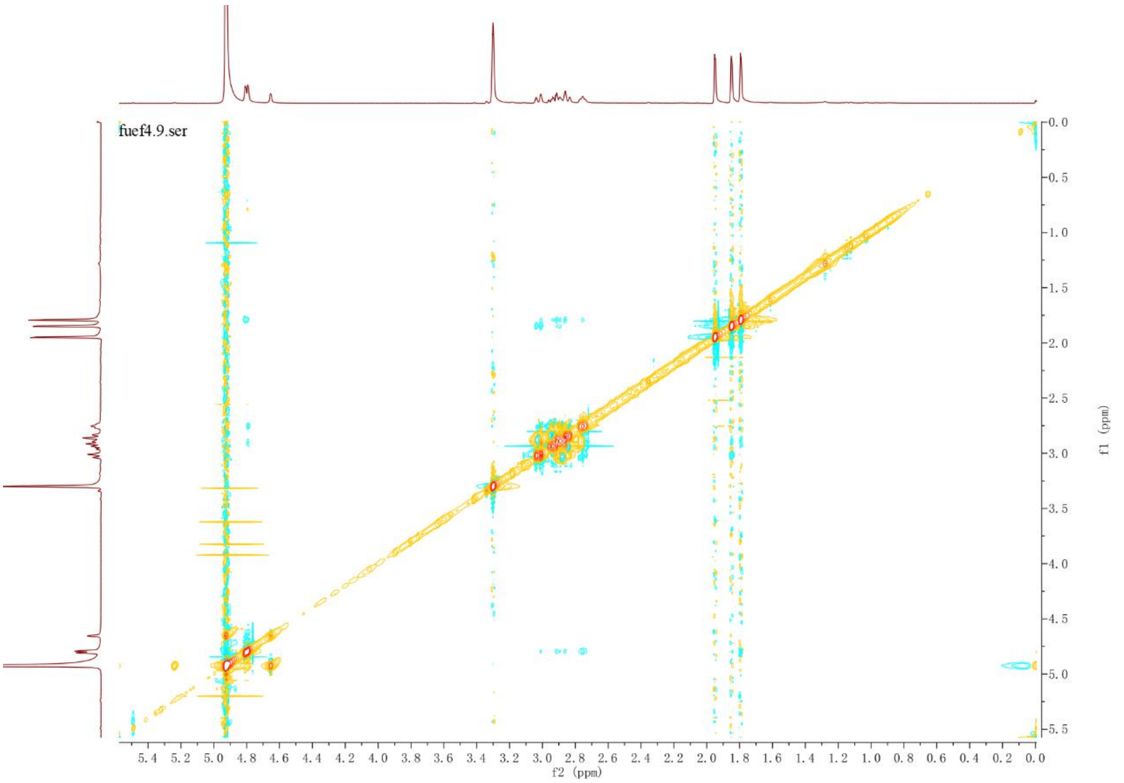

Figure S13. NOESY spectrum of compound **2.**

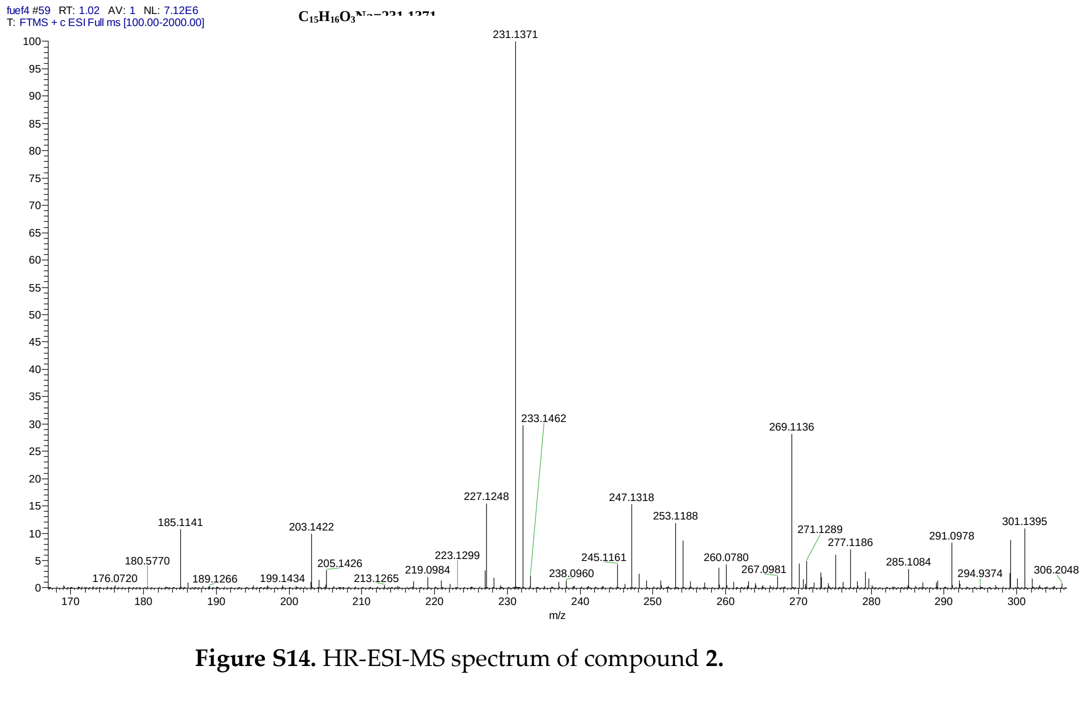

Figure S14. HR-ESI-MS spectrum of compound **2.**

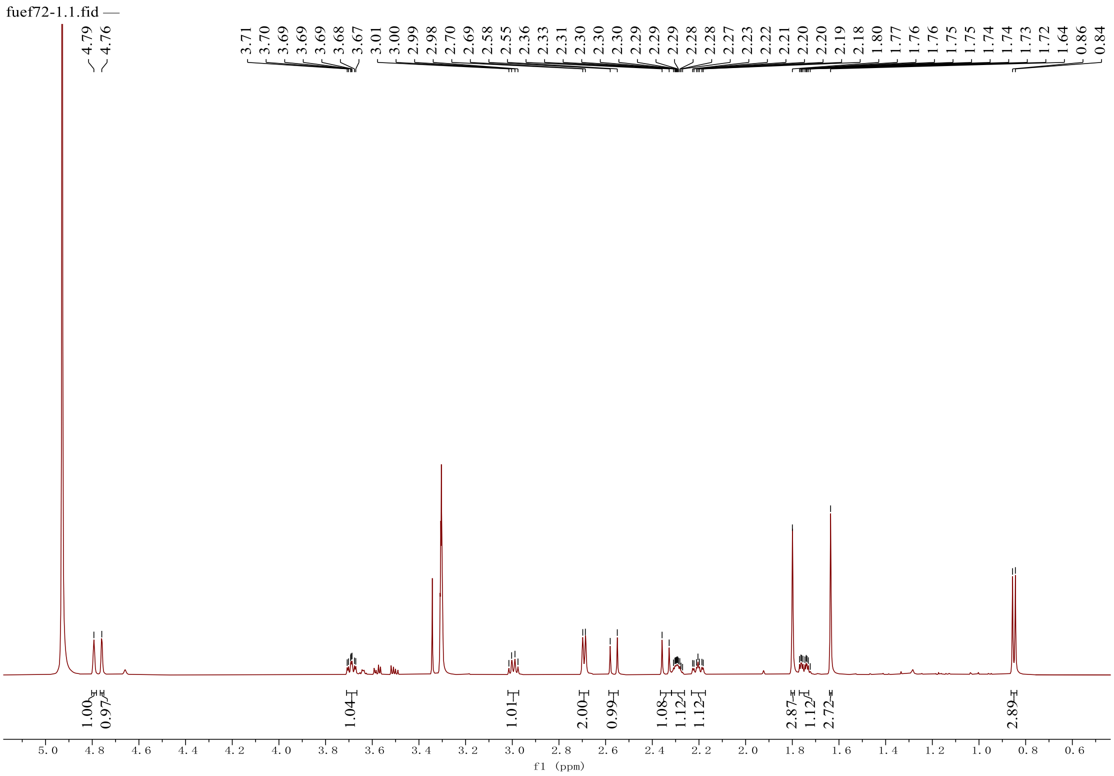

Figure S15. ^{1}H-NMR spectrum (600 MHz, CD_3OD) of compound **3.**

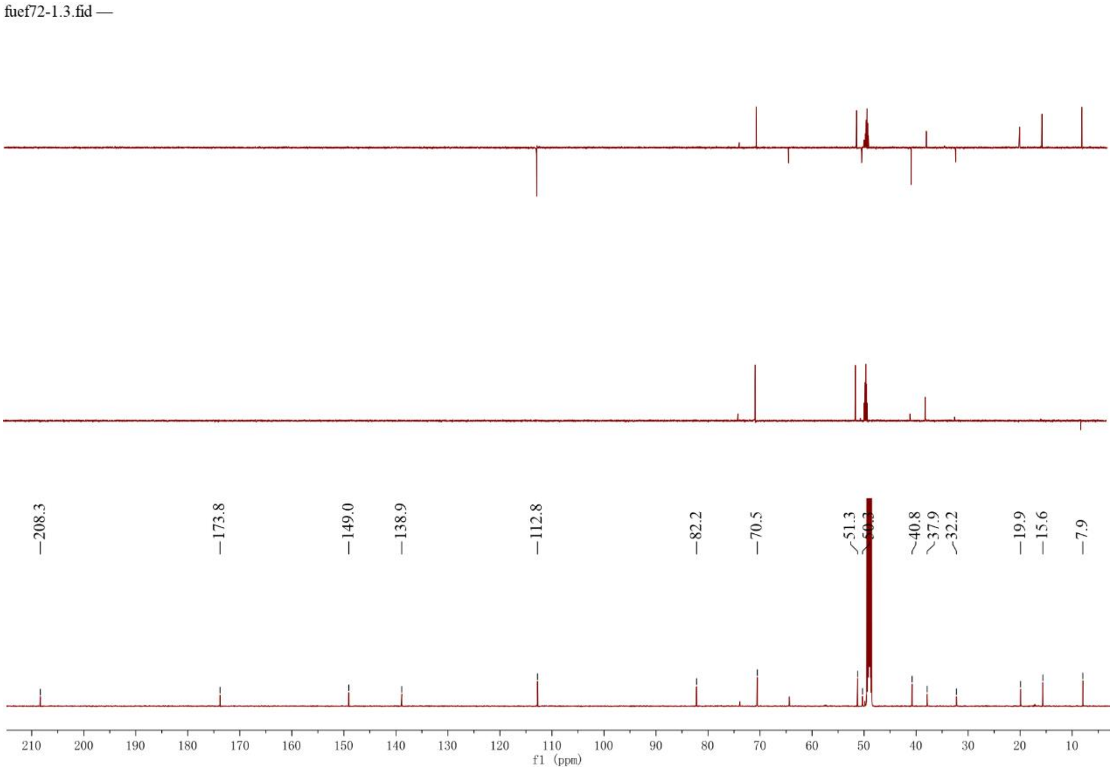

Figure S16. ^{13}C-NMR spectrum (150 MHz, CD_3OD) of compound **3.**

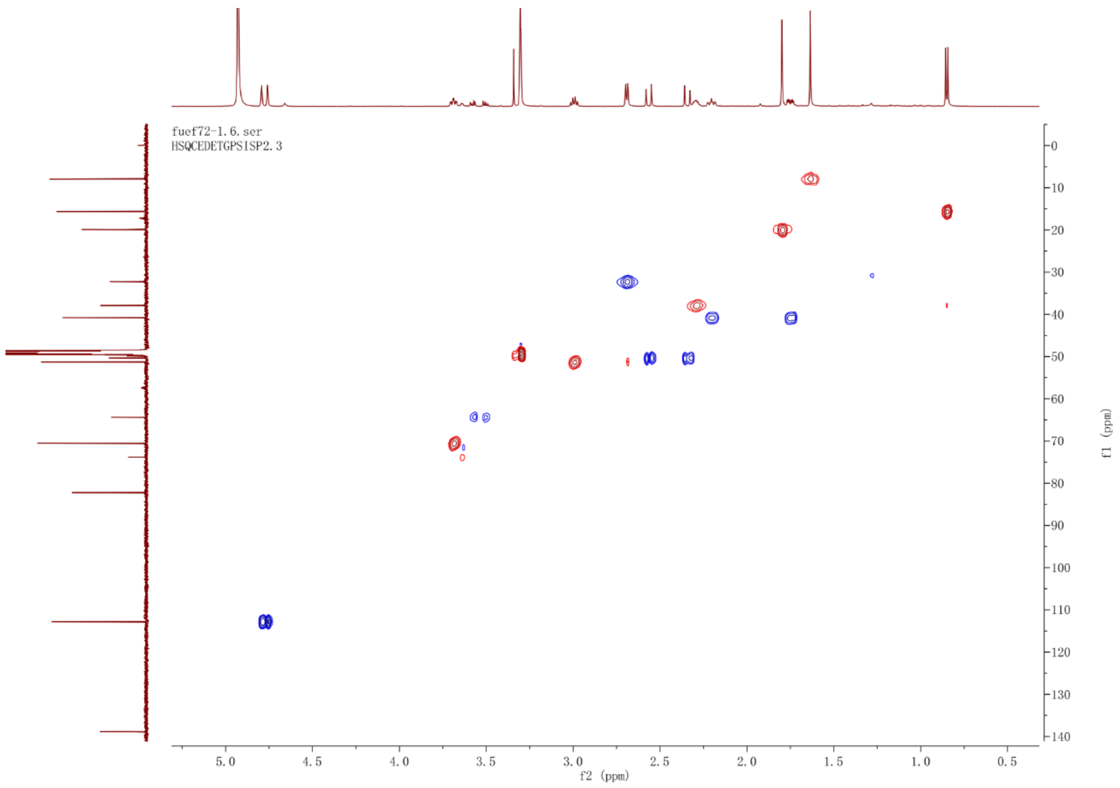

Figure S17. HSQC spectrum of compound **3.**

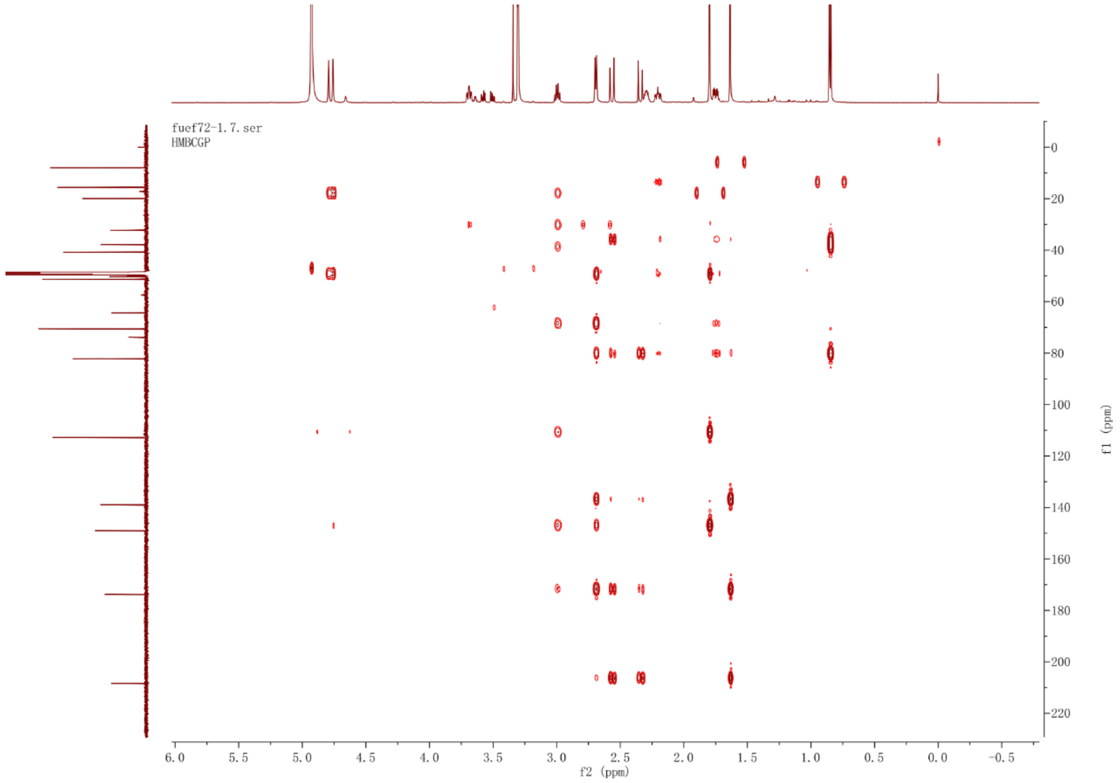

Figure S18. HMBC spectrum of compound **3.**

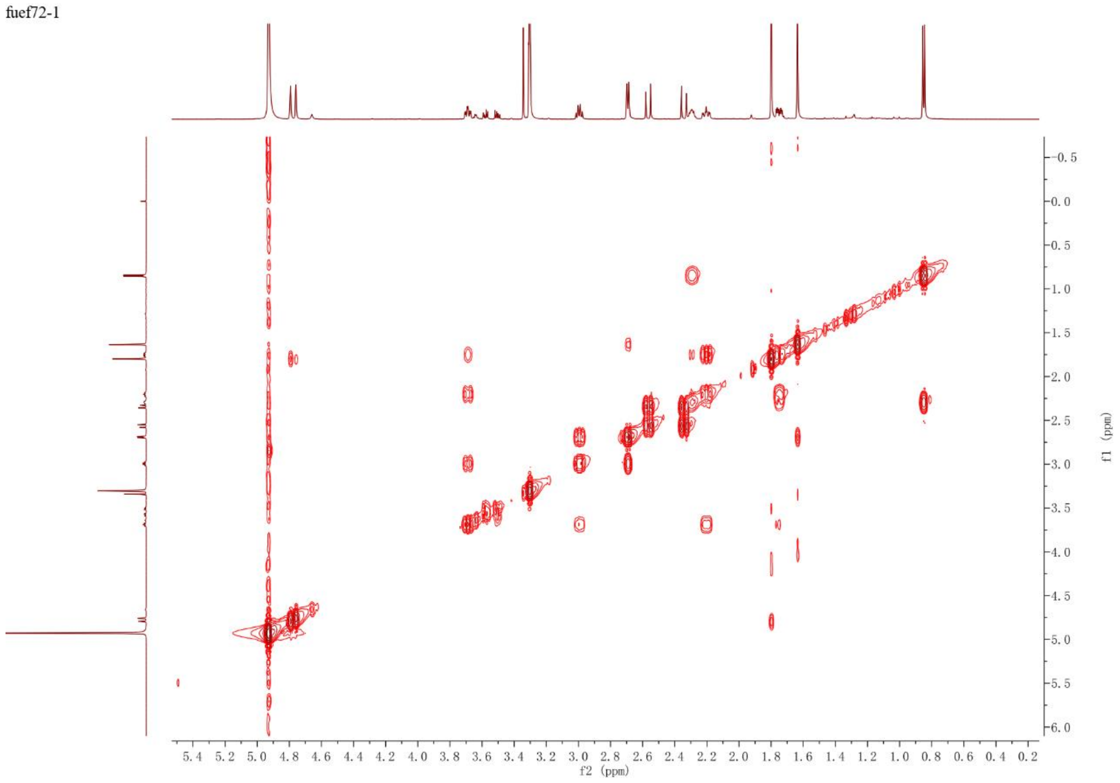

Figure S19. COSY spectrum of compound **3.**

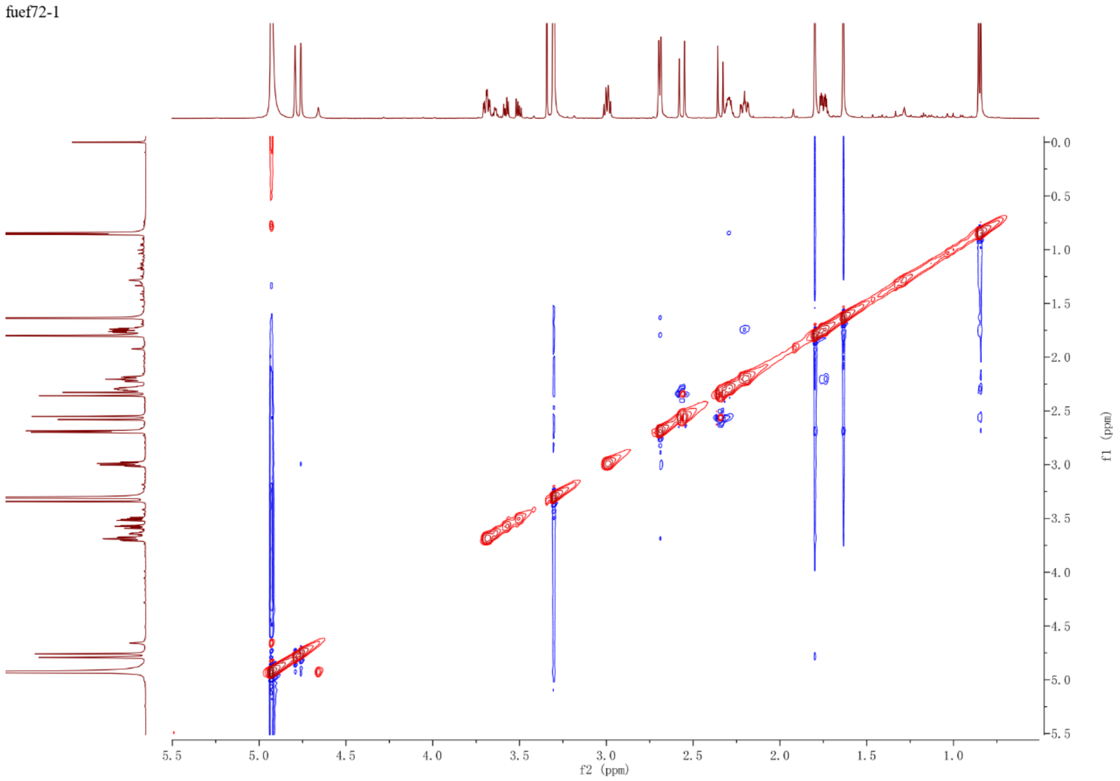

Figure S20. NOESY spectrum of compound **3.**

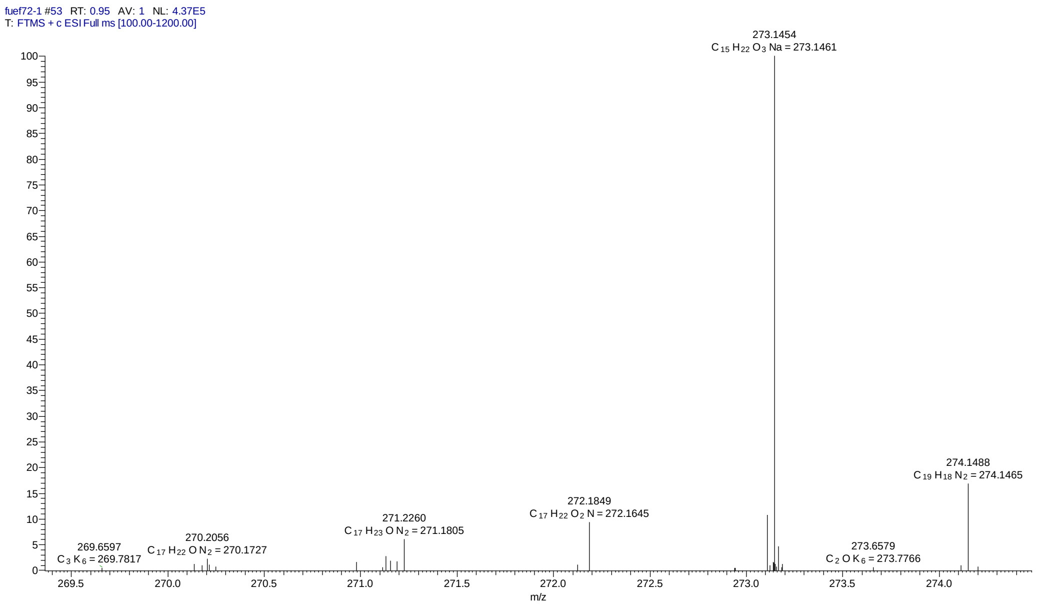

Figure S21. HR-ESI-MS spectrum of compound **3.**

The energy distribution and boltzmann weights of each configuration of compounds 1 - 3

Table S1. The energy distribution and boltzmann weights of each configuration of compound (4S, 5S, 6R,7S, 8R, 10R, 11R)-1.

State	GEPOP	SEPOP	weighting factor
**1a**	26.2957	19.4299	24.35
**1b**	22.4009	19.7417	24.35
**1c**	17.5568	21.8005	0.30
**1d**	12.2816	10.0760	25.90
**1e**	5.9946	4.6663	0.21
**1f**	4.1302	2.6035	0.16
**1g**	2.1996	1.7563	24.21
**1h**	1.2025	3.3358	0.52
**total**	>90	>83	100

(The distribution details of each compound to see below).

Table S2. The energy distribution and boltzmann weights of each configuration of compound (6*S*)-**2.**

State	GEPOP	SEPOP	weighting factor
**2a**	40.5095	21.5857	9.70
**2b**	16.8445	18.1663	2.07
**2c**	14.8961	26.5200	14.78
**2d**	14.1869	19.4377	17.54
**2e**	7.5272	7.8536	5.85
**2f**	2.1944	1.0488	34.52
**2g**	1.8078	0.8340	15.28
**2h**	1.5303	3.6653	0.25
**total**	>99	>99	100

(The distribution details of each compound to see below).

Table S3. The energy distribution and boltzmann weights of each configuration of compound (6*R*, 7*R*, 9*S*, 10*R*)-**3.**

State	GEPOP	SEPOP	weighting factor
3a	25.9840	30.1491	2.79
3b	17.6164	28.6005	45.01
3c	14.2535	3.8509	1.32
3d	11.4058	19.5004	40.31
3e	8.7712	1.9112	2.17
3f	6.6433	7.0024	2.65
3g	4.5183	1.9994	1.43
3h	3.7690	1.8167	0.94
3i	2.8259	2.0964	1.18
3j	2.0853	0.4786	1.14
3k	1.0949	0.6066	1.06
total	>94	>97	100

(The distribution details of each compound to see below).

Compound 1

Conformer 1a

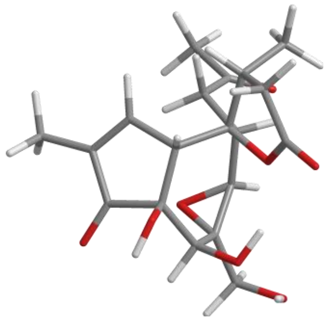

Table S4. Standard orientation of 1a.

Center Number	Atomic Number	Atomic Type	Coordinates (Angstroms) X	Y	Z
1	6	0	-0.503345	0.257830	-1.419654
2	6	0	-1.785199	-0.341141	-0.764922
3	6	0	-2.084930	-0.240835	0.752345
4	6	0	-1.010372	-0.725279	1.720082
5	6	0	0.444191	-0.528170	1.579039
6	6	0	1.272387	0.200598	0.523815
7	6	0	0.523996	1.025924	-0.540880
8	6	0	0.072263	-0.903154	-2.205223
9	6	0	-0.610198	-2.056081	-2.092741
10	6	0	-1.753555	-1.833930	-1.193043
11	8	0	-2.629784	-2.631299	-0.893148
12	6	0	-0.352953	-3.375387	-2.749010
13	8	0	-2.880858	0.252914	-1.475039
14	8	0	-2.596473	1.025255	1.168780
15	6	0	-1.546132	-0.936820	3.136090
16	8	0	-0.242475	2.046213	0.200727
17	6	0	-0.314261	3.229978	-0.486151
18	6	0	0.553130	3.135933	-1.721161
19	6	0	1.455113	1.917267	-1.445342

20	8	0	-0.996984	4.138032	-0.082282
21	8	0	-0.174774	-1.794748	1.232842
22	8	0	-1.862644	0.292573	3.775035
23	6	0	2.292233	-0.805980	-0.054229
24	6	0	3.465826	-1.079509	0.886698
25	6	0	4.387420	-2.210378	0.486592
26	8	0	3.650251	-0.421849	1.898383
27	6	0	2.760830	2.396006	-0.788238
28	1	0	-0.876340	0.990029	-2.145994
29	1	0	-2.932189	-0.926997	0.884807
30	1	0	0.998312	-0.598850	2.515519
31	1	0	1.846592	0.931942	1.100743
32	1	0	0.946727	-0.779085	-2.837497
33	1	0	-1.224597	-3.693019	-3.333313
34	1	0	0.515131	-3.331322	-3.412074
35	1	0	-0.176755	-4.152422	-1.995428
36	1	0	-3.685245	-0.234652	-1.222649
37	1	0	-1.884057	1.674726	1.009859
38	1	0	-2.421514	-1.605344	3.091022
39	1	0	-0.780141	-1.433051	3.739214
40	1	0	-0.097071	3.008713	-2.594029
41	1	0	1.105822	4.067014	-1.866836
42	1	0	1.692730	1.387595	-2.371668
43	1	0	-2.400176	0.789948	3.127906
44	1	0	2.727374	-0.457398	-0.998685
45	1	0	1.808979	-1.758592	-0.292475
46	1	0	3.856817	-3.165559	0.588023
47	1	0	4.685822	-2.120898	-0.563993
48	1	0	5.271311	-2.220282	1.127376
49	1	0	2.566259	2.970441	0.124805
50	1	0	3.443315	1.582548	-0.534491

Conformer 1b

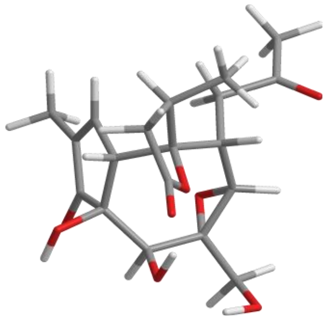

Table S5. Standard orientation of 1b.

Center Number	Atomic Number	Atomic Type	Coordinates (Angstroms) X	Y	Z
1	6	0	-0.503255	0.257048	-1.419911
2	6	0	-1.784434	-0.343406	-0.765303
3	6	0	-2.084862	-0.242636	0.751753
4	6	0	-1.010029	-0.725416	1.720066
5	6	0	0.444361	-0.526810	1.579578
6	6	0	1.272044	0.202157	0.524125
7	6	0	0.523049	1.026410	-0.540983
8	6	0	0.073856	-0.903364	-2.205228
9	6	0	-0.607027	-2.057181	-2.092424
10	6	0	-1.750649	-1.836364	-1.192711
11	8	0	-2.625720	-2.634843	-0.892454
12	6	0	-0.347960	-3.376280	-2.748401
13	8	0	-2.880637	0.248730	-1.476284
14	8	0	-2.597982	1.023083	1.167395
15	6	0	-1.546208	-0.936691	3.136016
16	8	0	-0.244652	2.046053	0.200155
17	6	0	-0.317416	3.229664	-0.486966
18	6	0	0.550259	3.136123	-1.721779
19	6	0	1.453554	1.918567	-1.445369
20	8	0	-1.001029	4.137131	-0.083295
21	8	0	-0.173225	-1.794292	1.233809
22	8	0	-1.864677	0.292631	3.774013
23	6	0	2.292692	-0.803803	-0.053508
24	6	0	3.466251	-1.076475	0.887726
25	6	0	4.388097	-2.207413	0.488447
26	8	0	3.650445	-0.418065	1.898955
27	6	0	2.758498	2.398949	-0.788015
28	1	0	-0.876966	0.988820	-2.146304
29	1	0	-2.931317	-0.929785	0.884298
30	1	0	0.998158	-0.596521	2.516289
31	1	0	1.845615	0.934248	1.100755
32	1	0	0.948103	-0.778271	-2.837601
33	1	0	-1.219310	-3.695439	-3.332305
34	1	0	0.519821	-3.331064	-3.411795
35	1	0	-0.170273	-4.152844	-1.994679
36	1	0	-3.684555	-0.239475	-1.223654
37	1	0	-1.886046	1.673171	1.008844
38	1	0	-2.420664	-1.606465	3.090908
39	1	0	-0.779875	-1.431548	3.739837
40	1	0	-0.099688	3.007740	-2.594678
41	1	0	1.101881	4.067769	-1.867858

42	1	0	1.692038	1.388880	-2.371456
43	1	0	-2.402506	0.788974	3.126340
44	1	0	2.727958	-0.454936	-0.997815
45	1	0	1.810194	-1.756729	-0.291980
46	1	0	3.857935	-3.162679	0.591382
47	1	0	4.685853	-2.119178	-0.562428
48	1	0	5.272372	-2.216207	1.128730
49	1	0	2.563106	2.973634	0.124696
50	1	0	3.441706	1.586274	-0.533667
51	1	0	3.281360	3.059716	-1.486872

Conformer 1c

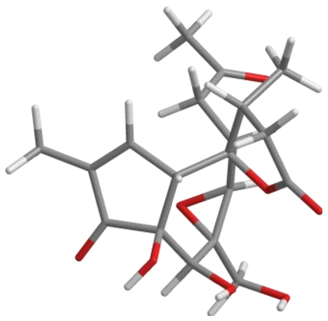

Table S6. Standard orientation of 1c.

Center Number	Atomic Number	Atomic Type	Coordinates (Angstroms) X	Y	Z
1	6	0	-0.526063	0.097747	-1.423962
2	6	0	-1.747371	-0.553209	-0.711442
3	6	0	-2.058753	-0.373888	0.794829
4	6	0	-0.955158	-0.566703	1.818436
5	6	0	0.482130	-0.300338	1.644940
6	6	0	1.267730	0.318385	0.491613
7	6	0	0.460138	1.004117	-0.628112
8	6	0	0.134229	-1.072063	-2.125062
9	6	0	-0.454247	-2.265086	-1.923562
10	6	0	-1.625256	-2.058586	-1.063978
11	8	0	-2.474711	-2.879595	-0.748000
12	6	0	-0.092720	-3.606214	-2.478727
13	8	0	-2.927504	-0.074547	-1.397667
14	8	0	-2.704170	0.864965	1.064783
15	6	0	-1.486369	-0.559893	3.252417
16	8	0	-0.340487	2.028486	0.042606
17	6	0	-0.480144	3.142955	-0.721368
18	6	0	0.351314	2.994925	-1.981109
19	6	0	1.325531	1.852739	-1.635758

20	8	0	-1.174025	4.069076	-0.371793
21	8	0	-0.051840	-1.645903	1.503307
22	8	0	-1.808395	0.753776	3.688465
23	6	0	2.315708	-0.707283	0.009936
24	6	0	3.508695	-0.837337	0.956828
25	6	0	4.458722	-1.980309	0.673565
26	8	0	3.686353	-0.064589	1.884980
27	6	0	2.622103	2.447602	-1.061817
28	1	0	-0.960790	0.740979	-2.198844
29	1	0	-2.775393	-1.190014	0.994471
30	1	0	1.028781	-0.192448	2.582203
31	1	0	1.825287	1.129725	0.968955
32	1	0	0.998118	-0.931183	-2.767799
33	1	0	-0.941081	-4.040801	-3.020134
34	1	0	0.760837	-3.541412	-3.158591
35	1	0	0.158544	-4.303430	-1.670346
36	1	0	-3.610493	-0.765222	-1.300769
37	1	0	-3.276981	1.033903	0.293107
38	1	0	-2.355947	-1.233167	3.322099
39	1	0	-0.711887	-0.944978	3.921999
40	1	0	-0.313991	2.760684	-2.819900
41	1	0	0.847608	3.938575	-2.219887
42	1	0	1.568478	1.259228	-2.520994
43	1	0	-2.396209	1.122693	3.002684
44	1	0	2.728661	-0.443895	-0.971837
45	1	0	1.867681	-1.697325	-0.118573
46	1	0	3.965230	-2.929982	0.916736
47	1	0	4.721978	-2.020356	-0.389268
48	1	0	5.360867	-1.879885	1.280282
49	1	0	2.423460	3.086581	-0.193573
50	1	0	3.351066	1.691025	-0.763487
51	1	0	3.093101	3.071988	-1.828007

Conformer 1d

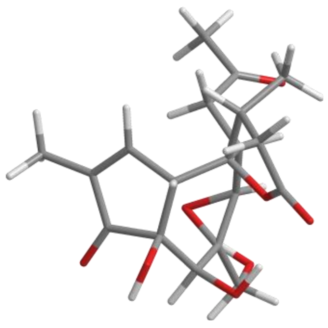

Table S7. Standard orientation of 1d.

Center Number	Atomic Number	Atomic Type	Coordinates (Angstroms) X	Y	Z
1	6	0	0.667165	0.827696	1.158283
2	6	0	1.933230	0.172196	0.526211
3	6	0	2.044458	-0.175750	-0.982734
4	6	0	0.940273	-1.039302	-1.578872
5	6	0	-0.501957	-0.938791	-1.294460
6	6	0	-1.294176	0.000278	-0.390067
7	6	0	-0.549504	1.183417	0.257338
8	6	0	0.345178	-0.075554	2.331871
9	6	0	1.157242	-1.138541	2.466420
10	6	0	2.149431	-1.106685	1.379355
11	8	0	3.081685	-1.877571	1.203479
12	6	0	1.154803	-2.203717	3.516552
13	8	0	3.014037	1.056407	0.857446
14	8	0	2.323127	0.927774	-1.826221
15	6	0	1.349792	-1.679938	-2.909130
16	8	0	-0.022536	1.987809	-0.861606
17	6	0	-0.032659	3.327038	-0.576812
18	6	0	-0.729330	3.543152	0.748333
19	6	0	-1.483084	2.220434	0.986764
20	8	0	0.465474	4.124681	-1.331852
21	8	0	0.311386	-1.981102	-0.679035
22	8	0	0.430742	-2.668196	-3.339164
23	6	0	-2.095659	-0.869196	0.604242
24	6	0	-3.323188	-1.526974	-0.026366
25	6	0	-4.013162	-2.582718	0.809124
26	8	0	-3.725142	-1.216388	-1.136322
27	6	0	-2.908299	2.344715	0.421814
28	1	0	1.020824	1.781405	1.568470
29	1	0	2.955486	-0.788860	-1.025302
30	1	0	-1.135485	-1.356154	-2.074997
31	1	0	-2.025191	0.457164	-1.064170
32	1	0	-0.460067	0.159422	3.022139
33	1	0	2.125878	-2.248801	4.023549
34	1	0	0.377428	-2.028112	4.264930
35	1	0	0.987587	-3.189236	3.065267
36	1	0	3.840670	0.602011	0.616042
37	1	0	1.540872	1.508473	-1.789269
38	1	0	1.396400	-0.913537	-3.687140
39	1	0	2.360073	-2.104179	-2.793940
40	1	0	0.028507	3.756185	1.510439
41	1	0	-1.383109	4.416784	0.693696

42	1	0	-1.539256	1.987140	2.053259
43	1	0	0.284572	-3.246008	-2.568933
44	1	0	-2.462892	-0.287188	1.458432
45	1	0	-1.464276	-1.651213	1.036772
46	1	0	-3.378835	-3.477077	0.857475
47	1	0	-4.158874	-2.238382	1.838985
48	1	0	-4.973214	-2.848957	0.362499
49	1	0	-2.899087	2.617572	-0.639777
50	1	0	-3.499468	1.433183	0.529480
51	1	0	-3.432060	3.140498	0.961105

Conformer 1e

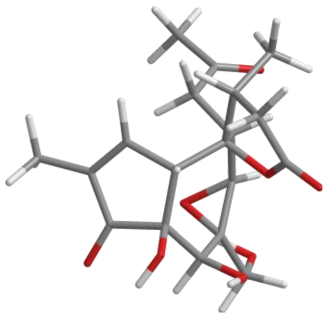

Table S8. Standard orientation of 1e.

Center	Atomic	Atomic	Coordinates (Angstroms)		
Number	Number	Type	X	Y	Z
1	6	0	-0.710499	0.788780	-1.146234
2	6	0	-1.932544	0.054849	-0.519549
3	6	0	-2.030097	-0.337848	0.978307
4	6	0	-0.859687	-1.050969	1.625481
5	6	0	0.570332	-0.880052	1.325886
6	6	0	1.306326	0.062201	0.378967
7	6	0	0.498570	1.208792	-0.259265
8	6	0	-0.342453	-0.086622	-2.327124
9	6	0	-1.102295	-1.186042	-2.480997
10	6	0	-2.116046	-1.194098	-1.419796
11	8	0	-3.053802	-1.968374	-1.289543
12	6	0	-1.043205	-2.238632	-3.542130
13	8	0	-3.081543	0.899310	-0.761749
14	8	0	-2.398682	0.752601	1.803852
15	6	0	-1.221793	-1.641917	2.992807
16	8	0	-0.036975	1.980757	0.862068
17	6	0	-0.082470	3.309550	0.585423
18	6	0	0.559364	3.563005	-0.765301
19	6	0	1.368783	2.278061	-1.022494

**20**	**8**	**0**	**-0.570068**	**4.102190**	**1.357102**
**21**	**8**	**0**	**-0.185559**	**-1.999132**	**0.766196**
**22**	**8**	**0**	**-0.255967**	**-2.572775**	**3.448673**
**23**	**6**	**0**	**2.119331**	**-0.794624**	**-0.614927**
**24**	**6**	**0**	**3.379980**	**-1.399180**	**0.003348**
**25**	**6**	**0**	**4.094102**	**-2.440088**	**-0.830876**
**26**	**8**	**0**	**3.789430**	**-1.059863**	**1.102068**
**27**	**6**	**0**	**2.800575**	**2.467879**	**-0.494176**
**28**	**1**	**0**	**-1.120244**	**1.723158**	**-1.549123**
**29**	**1**	**0**	**-2.852003**	**-1.076861**	**0.977607**
**30**	**1**	**0**	**1.226744**	**-1.227183**	**2.121685**
**31**	**1**	**0**	**2.034734**	**0.559523**	**1.026765**
**32**	**1**	**0**	**0.455396**	**0.191441**	**-3.009415**
**33**	**1**	**0**	**-2.013019**	**-2.334888**	**-4.043965**
**34**	**1**	**0**	**-0.281468**	**-2.009324**	**-4.291924**
**35**	**1**	**0**	**-0.814857**	**-3.217247**	**-3.102785**
**36**	**1**	**0**	**-3.862080**	**0.313501**	**-0.781757**
**37**	**1**	**0**	**-2.981566**	**1.300739**	**1.245575**
**38**	**1**	**0**	**-1.281064**	**-0.840000**	**3.733873**
**39**	**1**	**0**	**-2.216884**	**-2.108945**	**2.920723**
**40**	**1**	**0**	**-0.229360**	**3.735832**	**-1.506208**
**41**	**1**	**0**	**1.170600**	**4.467910**	**-0.730422**
**42**	**1**	**0**	**1.410595**	**2.042865**	**-2.089278**
**43**	**1**	**0**	**-0.083198**	**-3.163428**	**2.693559**
**44**	**1**	**0**	**2.452019**	**-0.212600**	**-1.483514**
**45**	**1**	**0**	**1.510704**	**-1.606347**	**-1.024797**
**46**	**1**	**0**	**3.485744**	**-3.352595**	**-0.870922**
**47**	**1**	**0**	**4.222299**	**-2.096537**	**-1.863401**
**48**	**1**	**0**	**5.064995**	**-2.675401**	**-0.390373**
**49**	**1**	**0**	**2.805322**	**2.745729**	**0.566198**
**50**	**1**	**0**	**3.427929**	**1.581773**	**-0.612265**
**51**	**1**	**0**	**3.276347**	**3.282885**	**-1.049280**

Conformer 1f

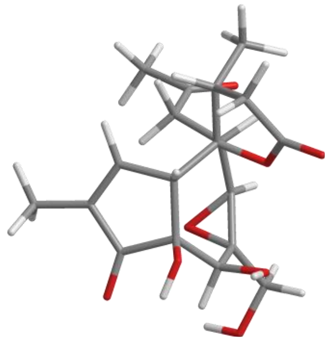

Table S9. Standard orientation of 1f.

Center Number	Atomic Number	Atomic Type	Coordinates (Angstroms) X	Y	Z
1	6	0	0.198023	0.826749	1.248088
2	6	0	1.657530	0.608600	0.749137
3	6	0	2.044041	0.451656	-0.742967
4	6	0	1.270279	-0.539614	-1.591126
5	6	0	-0.154630	-0.898670	-1.473465
6	6	0	-1.272309	-0.354299	-0.586868
7	6	0	-0.980142	0.917594	0.232535
8	6	0	0.025239	-0.244638	2.305947
9	6	0	1.090416	-1.046448	2.484616
10	6	0	2.153451	-0.607225	1.572189
11	8	0	3.304253	-1.017801	1.516858
12	6	0	1.278367	-2.172078	3.451674
13	8	0	2.416589	1.747575	1.219741
14	8	0	2.090672	1.694602	-1.423345
15	6	0	1.951693	-0.844761	-2.920903
16	8	0	-0.628560	1.941888	-0.750893
17	6	0	-1.068979	3.171574	-0.380124
18	6	0	-1.897162	3.047663	0.884457
19	6	0	-2.240687	1.547154	0.939537
20	8	0	-0.803883	4.161345	-1.021927
21	8	0	0.870481	-1.736622	-0.877561
22	8	0	3.258464	-1.379415	-2.738787
23	6	0	-1.851158	-1.532215	0.226659
24	6	0	-2.746829	-2.453789	-0.600711
25	6	0	-3.174745	-3.741139	0.069425
26	8	0	-3.099098	-2.168010	-1.734106
27	6	0	-3.585736	1.310828	0.232108
28	1	0	0.218547	1.794572	1.763725
29	1	0	3.063979	0.035217	-0.693246
30	1	0	-0.575169	-1.365970	-2.364619
31	1	0	-2.047652	-0.057599	-1.299719
32	1	0	-0.886339	-0.319469	2.891504
33	1	0	2.160482	-1.998368	4.078940
34	1	0	0.406320	-2.294655	4.099423
35	1	0	1.449896	-3.114670	2.917828
36	1	0	3.344499	1.454381	1.294808
37	1	0	2.382491	2.335001	-0.748175
38	1	0	1.322349	-1.533719	-3.500220
39	1	0	2.071681	0.079686	-3.491771
40	1	0	-1.298150	3.389009	1.736292
41	1	0	-2.773788	3.697375	0.828070

42	1	0	-2.314469	1.195670	1.972007
43	1	0	3.154117	-2.210664	-2.246014
44	1	0	-2.459330	-1.188121	1.072093
45	1	0	-1.051858	-2.133667	0.670395
46	1	0	-2.306299	-4.404545	0.170201
47	1	0	-3.551331	-3.552301	1.080957
48	1	0	-3.939966	-4.240505	-0.528202
49	1	0	-3.571117	1.685702	-0.797877
50	1	0	-3.882414	0.260242	0.203225
51	1	0	-4.367787	1.857247	0.769268

Conformer 1g

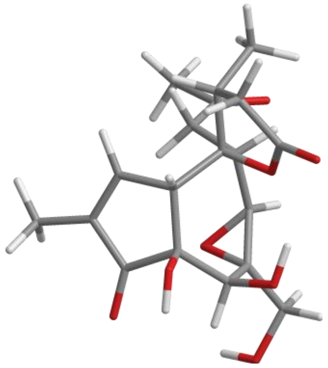

Table S10. Standard orientation of 1g.

Center Number	Atomic Number	Atomic Type	Coordinates (Angstroms) X	Y	Z
1	6	0	0.161059	0.842185	1.249515
2	6	0	1.627593	0.728747	0.729343
3	6	0	1.984658	0.615637	-0.775484
4	6	0	1.311692	-0.505047	-1.559518
5	6	0	-0.092623	-0.942911	-1.437698
6	6	0	-1.246168	-0.423390	-0.582152
7	6	0	-1.020557	0.861991	0.237431
8	6	0	0.072120	-0.240062	2.306992
9	6	0	1.188384	-0.973119	2.459971
10	6	0	2.198460	-0.489103	1.503725
11	8	0	3.351130	-0.878493	1.384712
12	6	0	1.465184	-2.084875	3.421684
13	8	0	2.298098	1.882436	1.260247
14	8	0	1.926577	1.838470	-1.488347
15	6	0	2.036301	-0.842503	-2.856721
16	8	0	-0.715593	1.916902	-0.747335
17	6	0	-1.216848	3.132410	-0.365842

18	6	0	-2.053821	2.950274	0.880722
19	6	0	-2.317364	1.432548	0.926127
20	8	0	-0.967587	4.130509	-0.995305
21	8	0	0.969703	-1.685570	-0.789986
22	8	0	3.368562	-1.282559	-2.614449
23	6	0	-1.787587	-1.613845	0.240959
24	6	0	-2.640587	-2.577708	-0.583535
25	6	0	-3.029949	-3.870004	0.099835
26	8	0	-2.989971	-2.318834	-1.724212
27	6	0	-3.642581	1.131722	0.205142
28	1	0	0.122326	1.808152	1.767358
29	1	0	3.049488	0.351153	-0.766703
30	1	0	-0.479375	-1.475904	-2.307033
31	1	0	-2.021425	-0.166750	-1.310673
32	1	0	-0.821249	-0.369409	2.911270
33	1	0	2.346032	-1.856631	4.033309
34	1	0	0.615016	-2.262888	4.085664
35	1	0	1.686568	-3.014307	2.883046
36	1	0	3.246922	1.772791	1.071415
37	1	0	0.989482	2.105567	-1.505366
38	1	0	1.464267	-1.601193	-3.407679
39	1	0	2.114507	0.054520	-3.476192
40	1	0	-1.484661	3.320050	1.740898
41	1	0	-2.963450	3.551924	0.816340
42	1	0	-2.381479	1.075991	1.957532
43	1	0	3.303748	-2.082323	-2.065753
44	1	0	-2.413632	-1.284909	1.078921
45	1	0	-0.968477	-2.178507	0.696838
46	1	0	-2.137701	-4.494746	0.233433
47	1	0	-3.438087	-3.678523	1.098653
48	1	0	-3.760165	-4.410689	-0.505519
49	1	0	-3.637900	1.511192	-0.823170
50	1	0	-3.887308	0.068329	0.170092
51	1	0	-4.455005	1.636533	0.737726

Conformer 1h

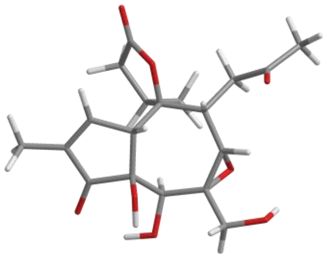

Table S11. Standard orientation of 1h.

Center Number	Atomic Number	Atomic Type	Coordinates (Angstroms) X	Y	Z
1	6	0	-1.002286	-0.409853	-0.940977
2	6	0	-1.674136	0.910842	-0.469783
3	6	0	-1.027890	1.759851	0.644494
4	6	0	0.382739	2.245363	0.302270
5	6	0	1.460638	1.325197	-0.091215
6	6	0	1.453205	-0.176769	-0.328390
7	6	0	0.160867	-1.008984	-0.072473
8	6	0	-2.176337	-1.354399	-1.088176
9	6	0	-3.345701	-0.887602	-0.609648
10	6	0	-3.110625	0.459554	-0.073281
11	8	0	-3.913495	1.154744	0.544797
12	6	0	-4.689406	-1.544127	-0.578568
13	8	0	-1.819402	1.733632	-1.635500
14	8	0	-1.853164	2.912558	0.834960
15	6	0	0.511641	3.711093	-0.123645
16	8	0	0.484379	-2.288273	-0.719632
17	6	0	0.082335	-3.370171	-0.003209
18	6	0	-0.606965	-2.916051	1.261434
19	6	0	-0.306215	-1.408272	1.381300
20	8	0	0.274461	-4.494946	-0.402298
21	8	0	1.407668	1.864441	1.257080
22	8	0	1.867416	4.101241	-0.273799
23	6	0	2.755557	-0.778577	0.239443
24	6	0	3.975252	-0.517922	-0.643836
25	6	0	5.314170	-0.905342	-0.053853
26	8	0	3.880338	-0.030941	-1.759540
27	6	0	0.648436	-1.121057	2.548954
28	1	0	-0.570094	-0.225717	-1.931238
29	1	0	-0.969614	1.187819	1.576259
30	1	0	2.255178	1.792312	-0.670379
31	1	0	1.569229	-0.248478	-1.417552
32	1	0	-2.058027	-2.326886	-1.556154
33	1	0	-5.419696	-0.958260	-1.149264
34	1	0	-4.650001	-2.553586	-0.995774
35	1	0	-5.064341	-1.603991	0.449764
36	1	0	-2.181767	2.578413	-1.305759
37	1	0	-2.753462	2.567663	1.019375
38	1	0	0.023602	3.854956	-1.091718
39	1	0	-0.000504	4.339504	0.613848
40	1	0	-1.676812	-3.129132	1.158121
41	1	0	-0.245404	-3.499257	2.112914
42	1	0	-1.241130	-0.883823	1.591051

43	1	0	2.309461	3.844505	0.554731
44	1	0	2.675129	-1.868785	0.326700
45	1	0	2.977049	-0.409364	1.245606
46	1	0	5.559763	-0.222995	0.769883
47	1	0	5.280806	-1.916203	0.367836
48	1	0	6.093758	-0.842746	-0.815739
49	1	0	1.547242	-1.743115	2.507963
50	1	0	0.950321	-0.071715	2.580109
51	1	0	0.132038	-1.357417	3.485833

Conformer 2

Conformer 2a

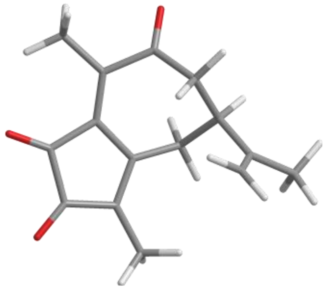

Table S12. Standard orientation of 2a.

Center Number	Atomic Number	Atomic Type	Coordinates (Angstroms) X	Y	Z
1	6	0	-1.250013	0.448757	-0.138839
2	6	0	-0.250297	-0.574036	-0.511359
3	6	0	1.043707	-0.206006	-1.173511
4	6	0	2.091160	0.525702	-0.279167
5	6	0	1.436608	1.600300	0.622045
6	6	0	0.327025	2.383572	-0.065071
7	6	0	-1.060403	1.790024	-0.034588
8	6	0	-2.508904	-0.301677	0.144259
9	6	0	-2.109790	-1.799562	0.117626
10	6	0	-0.717335	-1.850874	-0.334852
11	8	0	-3.632718	0.098046	0.375966
12	8	0	-2.861980	-2.717977	0.400572
13	6	0	-2.161430	2.795330	0.156553
14	6	0	2.961233	-0.473434	0.482154
15	6	0	4.153141	-0.982268	-0.294710
16	6	0	2.703939	-0.902817	1.722590
17	8	0	0.513429	3.497622	-0.534441
18	6	0	-0.023005	-3.143886	-0.626047
19	1	0	0.787958	0.464786	-2.006097
20	1	0	1.500697	-1.093337	-1.618772
21	1	0	2.746703	1.056016	-0.980357

22	1	0	1.008444	1.132201	1.513195
23	1	0	2.199385	2.313955	0.945228
24	1	0	-3.146433	2.333891	0.135757
25	1	0	-2.034214	3.331280	1.106090
26	1	0	-2.085414	3.549960	-0.635018
27	1	0	4.848775	-0.161940	-0.517752
28	1	0	4.697267	-1.752969	0.258914
29	1	0	3.853396	-1.406254	-1.262403
30	1	0	3.345502	-1.637927	2.201904
31	1	0	1.862532	-0.552282	2.312673
32	1	0	0.011577	-3.345835	-1.704627
33	1	0	1.006211	-3.140760	-0.254953
34	1	0	-0.566049	-3.967078	-0.153040

Conformer 2b

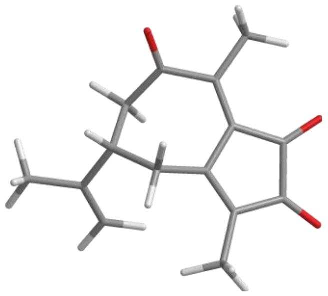

Table S13. Standard orientation of 2b.

Center	Atomic	Atomic	Coordinates (Angstroms)		
Number	Number	Type	X	Y	Z
1	6	0	1.150150	0.637658	0.065059
2	6	0	0.424948	-0.585834	0.481269
3	6	0	-0.906991	-0.533743	1.175253
4	6	0	-2.135675	0.031710	0.408843
5	6	0	-1.759322	1.218606	-0.523293
6	6	0	-0.797050	2.204158	0.116441
7	6	0	0.676536	1.909652	-0.006469
8	6	0	2.539660	0.195795	-0.257666
9	6	0	2.522944	-1.346517	-0.147279
10	6	0	1.197936	-1.713615	0.356099
11	8	0	3.521643	0.841725	-0.567153
12	8	0	3.470184	-2.068144	-0.415266
13	6	0	1.528852	3.127905	-0.230446
14	6	0	-2.942573	-0.998206	-0.378394
15	6	0	-4.439135	-0.804409	-0.338114
16	6	0	-2.377028	-1.982787	-1.085205
17	8	0	-1.181146	3.242135	0.637697
18	6	0	0.878838	-3.123754	0.740682

19	1	0	-0.749634	0.117371	2.048113
20	1	0	-1.159196	-1.520102	1.570328
21	1	0	-2.796748	0.438300	1.183042
22	1	0	-1.305733	0.819536	-1.436182
23	1	0	-2.664237	1.767416	-0.799016
24	1	0	2.590792	2.893802	-0.237109
25	1	0	1.263499	3.612636	-1.179301
26	1	0	1.307528	3.855231	0.558963
27	1	0	-4.719486	0.203795	-0.671777
28	1	0	-4.957019	-1.528602	-0.973873
29	1	0	-4.819973	-0.910208	0.686670
30	1	0	-2.979066	-2.685368	-1.655878
31	1	0	-1.300995	-2.121812	-1.135004
32	1	0	1.123958	-3.306690	1.795486
33	1	0	-0.176818	-3.367975	0.597957
34	1	0	1.485141	-3.812774	0.144667

Conformer 2c

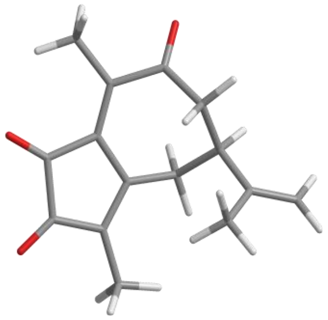

Table S14. Standard orientation of 2c.

Center Number	Atomic Number	Atomic Type	Coordinates (Angstroms) X	Y	Z
1	6	0	-1.158039	0.582850	-0.102701
2	6	0	-0.328646	-0.572242	-0.521037
3	6	0	0.997171	-0.388077	-1.200556
4	6	0	2.171848	0.242647	-0.388967
5	6	0	1.691137	1.373734	0.558550
6	6	0	0.664466	2.296620	-0.075153
7	6	0	-0.784490	1.885200	0.003742
8	6	0	-2.511070	0.025548	0.193419
9	6	0	-2.355993	-1.510269	0.098442
10	6	0	-0.999240	-1.763185	-0.392205
11	8	0	-3.553036	0.584966	0.474061
12	8	0	-3.236929	-2.312098	0.364204
13	6	0	-1.733728	3.028521	0.233767
14	6	0	3.014453	-0.791106	0.347280

15	6	0	2.402471	-1.540700	1.507353
16	6	0	4.272815	-1.022011	-0.045871
17	8	0	0.974113	3.380145	-0.551459
18	6	0	-0.562079	-3.147898	-0.745931
19	1	0	0.802160	0.284857	-2.048458
20	1	0	1.335783	-1.332417	-1.632528
21	1	0	2.820353	0.708989	-1.137814
22	1	0	1.250722	0.939744	1.462108
23	1	0	2.551016	1.981330	0.853995
24	1	0	-2.772807	2.707681	0.236254
25	1	0	-1.509294	3.528946	1.184786
26	1	0	-1.573260	3.775754	-0.551940
27	1	0	1.457477	-2.023306	1.230319
28	1	0	3.083133	-2.314868	1.873101
29	1	0	2.175978	-0.873819	2.349051
30	1	0	4.897078	-1.763061	0.447762
31	1	0	4.724399	-0.480560	-0.873913
32	1	0	-1.113927	-3.513132	-1.621733
33	1	0	0.505527	-3.214733	-0.963721
34	1	0	-0.793979	-3.833287	0.077487

Conformer 2d

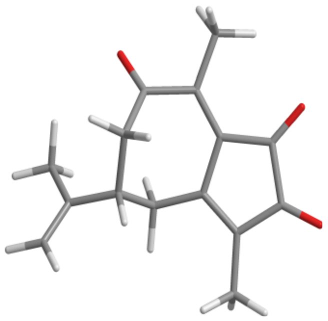

Table S15. Standard orientation of 2d.

Center Number	Atomic Number	Atomic Type	Coordinates (Angstroms) X	Y	Z
1	6	0	1.147261	0.660910	-0.072745
2	6	0	0.634370	-0.718261	-0.209906
3	6	0	-0.791269	-1.010061	-0.567456
4	6	0	-1.830461	-0.702367	0.542990
5	6	0	-1.643210	0.728246	1.146142
6	6	0	-1.068349	1.784961	0.213186
7	6	0	0.427341	1.809434	0.017449
8	6	0	2.634724	0.535300	-0.026444
9	6	0	2.919174	-0.985128	0.049660
10	6	0	1.628523	-1.658015	-0.110890

11	8	0	3.500651	1.387796	-0.025605
12	8	0	4.029410	-1.472048	0.191036
13	6	0	1.017797	3.191313	-0.039535
14	6	0	-3.250623	-0.939104	0.044664
15	6	0	-3.773423	-0.071503	-1.076076
16	6	0	-4.003248	-1.891728	0.607037
17	8	0	-1.762076	2.667259	-0.275743
18	6	0	1.523400	-3.145765	-0.231534
19	1	0	-0.895590	-2.057587	-0.861841
20	1	0	-1.028832	-0.405823	-1.454183
21	1	0	-1.650761	-1.409635	1.359033
22	1	0	-2.601831	1.102675	1.514895
23	1	0	-0.965423	0.648351	2.003318
24	1	0	0.814288	3.735638	0.891761
25	1	0	2.091050	3.171760	-0.215293
26	1	0	0.518409	3.751250	-0.838689
27	1	0	-3.217232	-0.233489	-2.008836
28	1	0	-4.826643	-0.287499	-1.277727
29	1	0	-3.678775	0.994469	-0.837258
30	1	0	-5.022694	-2.079826	0.278473
31	1	0	-3.630207	-2.516479	1.415341
32	1	0	2.421435	-3.611473	0.184640
33	1	0	0.647701	-3.540458	0.292002
34	1	0	1.449184	-3.455311	-1.282391

Conformer 2e

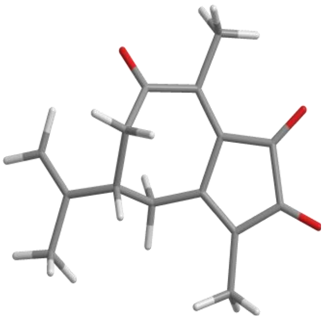

Table S16. Standard orientation of 2e.

Center	Atomic	Atomic	Coordinates (Angstroms)		
Number	Number	Type	X	Y	Z
1	6	0	1.162548	0.658266	-0.108958
2	6	0	0.605783	-0.709852	-0.181898
3	6	0	-0.828734	-0.965054	-0.537746
4	6	0	-1.874661	-0.584840	0.550588
5	6	0	-1.585740	0.818010	1.152973

6	6	0	-1.010366	1.853681	0.194894
7	6	0	0.478186	1.830304	-0.046620
8	6	0	2.645977	0.490490	-0.077461
9	6	0	2.884299	-1.032148	0.075157
10	6	0	1.572087	-1.673238	-0.042719
11	8	0	3.537379	1.314470	-0.131892
12	8	0	3.979301	-1.546913	0.233075
13	6	0	1.106229	3.190594	-0.173107
14	6	0	-3.282519	-0.768196	-0.010233
15	6	0	-3.765503	-2.198720	-0.079898
16	6	0	-4.046120	0.248906	-0.425215
17	8	0	-1.690986	2.755241	-0.277473
18	6	0	1.434617	-3.161246	-0.092742
19	1	0	-0.956737	-2.013782	-0.814073
20	1	0	-1.050382	-0.371746	-1.436150
21	1	0	-1.756289	-1.302576	1.372442
22	1	0	-2.500374	1.233421	1.582056
23	1	0	-0.862311	0.692502	1.965941
24	1	0	0.942433	3.777339	0.739926
25	1	0	2.173576	3.132160	-0.374551
26	1	0	0.603163	3.733497	-0.981704
27	1	0	-3.774468	-2.658108	0.917775
28	1	0	-4.776910	-2.257195	-0.492639
29	1	0	-3.112528	-2.822494	-0.704202
30	1	0	-5.039162	0.070363	-0.830810
31	1	0	-3.718074	1.283001	-0.383750
32	1	0	2.187788	-3.622520	0.554216
33	1	0	0.445103	-3.500397	0.222730
34	1	0	1.613135	-3.537592	-1.108979

Conformer 2f

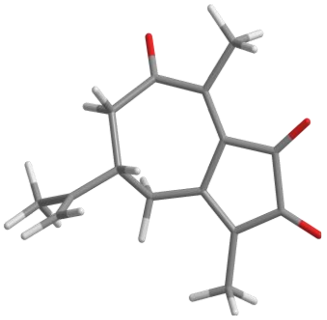

Table S17. Standard orientation of 2f.

Center	Atomic	Atomic	Coordinates (Angstroms)

Number	Number	Type	X	Y	Z
1	6	0	1.118663	0.573113	0.008509
2	6	0	0.510436	-0.721230	-0.373349
3	6	0	-0.942904	-0.877341	-0.693528
4	6	0	-1.839716	-0.170982	0.362830
5	6	0	-2.038597	1.309226	-0.003763
6	6	0	-0.823772	2.142979	-0.386107
7	6	0	0.578345	1.821953	0.061978
8	6	0	2.554797	0.275796	0.291987
9	6	0	2.731365	-1.237440	0.052663
10	6	0	1.422123	-1.748221	-0.352977
11	8	0	3.451223	1.022492	0.633599
12	8	0	3.782231	-1.844676	0.188287
13	6	0	1.400102	3.038342	0.428726
14	6	0	-3.177383	-0.881330	0.533573
15	6	0	-4.062622	-1.038681	-0.680974
16	6	0	-3.539437	-1.356107	1.730907
17	8	0	-0.993474	3.177844	-1.019600
18	6	0	1.236993	-3.197807	-0.665543
19	1	0	-1.188187	-1.940367	-0.744402
20	1	0	-1.146622	-0.462366	-1.691880
21	1	0	-1.325042	-0.222393	1.328830
22	1	0	-2.744722	1.418175	-0.833665
23	1	0	-2.492162	1.834110	0.849652
24	1	0	1.966733	2.866552	1.346136
25	1	0	2.132915	3.269979	-0.353472
26	1	0	0.753765	3.908031	0.551360
27	1	0	-3.543333	-1.560955	-1.494761
28	1	0	-4.963272	-1.609071	-0.436322
29	1	0	-4.381187	-0.069453	-1.084989
30	1	0	-4.487346	-1.868459	1.877459
31	1	0	-2.904125	-1.249252	2.607112
32	1	0	1.427707	-3.807532	0.226682
33	1	0	0.237443	-3.432471	-1.035316
34	1	0	1.967241	-3.513425	-1.420298

Conformer 2g

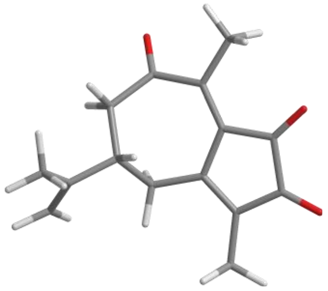

Table S18. Standard orientation of 2g.

Center Number	Atomic Number	Atomic Type	Coordinates (Angstroms) X	Y	Z
1	6	0	1.108276	0.581384	0.008429
2	6	0	0.557548	-0.739942	-0.356475
3	6	0	-0.874310	-0.987040	-0.715076
4	6	0	-1.846277	-0.247411	0.232909
5	6	0	-2.055730	1.204753	-0.237799
6	6	0	-0.848640	2.083820	-0.535836
7	6	0	0.535726	1.817250	-0.006621
8	6	0	2.540116	0.334903	0.359748
9	6	0	2.779045	-1.175046	0.149541
10	6	0	1.501197	-1.735179	-0.285897
11	8	0	3.393048	1.116091	0.733060
12	8	0	3.846309	-1.739742	0.333203
13	6	0	1.310181	3.063114	0.362878
14	6	0	-3.206214	-0.925714	0.378817
15	6	0	-3.931250	-0.646439	1.673801
16	6	0	-3.744010	-1.693013	-0.575879
17	8	0	-1.022648	3.108647	-1.184931
18	6	0	1.332515	-3.194054	-0.574514
19	1	0	-1.063232	-2.062711	-0.685634
20	1	0	-1.051091	-0.670937	-1.754499
21	1	0	-1.390010	-0.223427	1.231654
22	1	0	-2.673745	1.214159	-1.143053
23	1	0	-2.624648	1.762705	0.519264
24	1	0	1.737847	2.972353	1.364910
25	1	0	2.150885	3.227281	-0.320253
26	1	0	0.658868	3.936196	0.320122
27	1	0	-4.067005	0.430738	1.838317
28	1	0	-4.918880	-1.116539	1.689977
29	1	0	-3.356933	-1.022226	2.531332
30	1	0	-4.727615	-2.138672	-0.450589
31	1	0	-3.237598	-1.906427	-1.513062

32	1	0	2.278096	-3.712842	-0.395234
33	1	0	0.565325	-3.648613	0.063536
34	1	0	1.037023	-3.369318	-1.615591

Conformer 2h

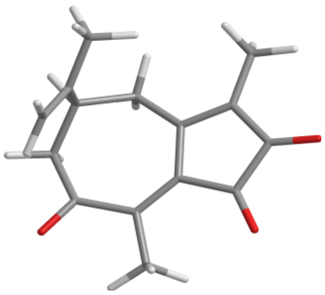

Table S19. Standard orientation of 2h.

Center Number	Atomic Number	Atomic Type	Coordinates (Angstroms) X	Y	Z
1	6	0	0.603863	-0.856537	-0.206748
2	6	0	0.666069	0.577525	-0.571974
3	6	0	-0.420556	1.363225	-1.239320
4	6	0	-1.859474	1.172608	-0.713644
5	6	0	-2.411658	-0.206741	-1.098238
6	6	0	-1.874949	-1.461062	-0.427469
7	6	0	-0.412521	-1.769706	-0.212416
8	6	0	1.987358	-1.194733	0.271700
9	6	0	2.802653	0.108610	0.207451
10	6	0	1.905149	1.124215	-0.333107
11	8	0	2.441882	-2.249471	0.667463
12	8	0	3.971020	0.212252	0.546847
13	6	0	-0.159478	-3.218269	0.144612
14	6	0	-2.039463	1.579352	0.745165
15	6	0	-1.541654	2.961163	1.107076
16	6	0	-2.652590	0.815541	1.657402
17	8	0	-2.688358	-2.309072	-0.070582
18	6	0	2.375680	2.518817	-0.604836
19	1	0	-0.417800	1.084790	-2.304163
20	1	0	-0.155059	2.422549	-1.212833
21	1	0	-2.454568	1.889292	-1.299784
22	1	0	-3.493493	-0.237586	-0.943561
23	1	0	-2.247673	-0.342994	-2.177227
24	1	0	0.759214	-3.586411	-0.311528
25	1	0	-0.047092	-3.344071	1.229011
26	1	0	-1.005966	-3.827160	-0.170700
27	1	0	-1.919792	3.716378	0.405075
28	1	0	-1.860506	3.242015	2.114831

29	1	0	-0.446461	3.023424	1.077865
30	1	0	-2.790963	1.171158	2.675583
31	1	0	-3.045113	-0.172186	1.442884
32	1	0	3.391600	2.640046	-0.219419
33	1	0	2.391490	2.736257	-1.679816
34	1	0	1.735242	3.270774	-0.129982

Conformer 3
Conformer 3a

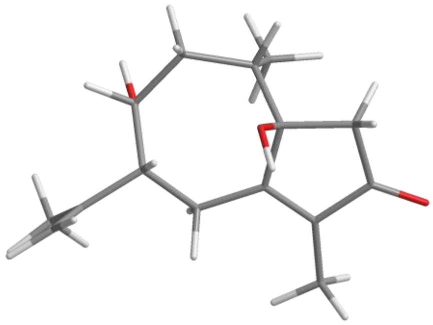

Table S20. Standard orientation of 3a.

Center Number	Atomic Number	Atomic Type	Coordinates (Angstroms) X	Y	Z
1	6	0	1.143600	0.714100	0.696700
2	6	0	0.784800	-0.591100	0.030300
3	6	0	-0.632800	-1.043600	-0.202400
4	6	0	-1.776100	-0.227900	0.439500
5	6	0	-1.956700	1.183200	-0.169700
6	6	0	-0.941200	2.220600	0.324200
7	6	0	0.558900	1.986400	0.030800
8	6	0	2.684900	0.738500	0.716600
9	6	0	3.043300	-0.545500	0.041000
10	6	0	1.851400	-1.313300	-0.352900
11	8	0	4.185800	-0.929600	-0.160700
12	6	0	-3.085800	-1.034600	0.386100
13	6	0	-3.703900	-1.315000	-0.958700
14	6	0	-3.667700	-1.486300	1.511700
15	6	0	0.878500	2.106200	-1.467100
16	8	0	-1.942400	1.137500	-1.596600
17	6	0	1.966600	-2.618200	-1.046100
18	8	0	0.703500	0.662000	2.056500
19	1	0	-0.789000	-1.103500	-1.287300
20	1	0	-0.707300	-2.074300	0.173600
21	1	0	-1.548900	-0.095300	1.505000
22	1	0	-2.949800	1.554900	0.116900

23	1	0	-1.210900	3.190100	-0.117600
24	1	0	-1.075000	2.346700	1.406400
25	1	0	1.057200	2.841300	0.513600
26	1	0	3.118800	1.566100	0.149700
27	1	0	3.091500	0.737300	1.733200
28	1	0	-4.572900	-1.977400	-0.875700
29	1	0	-4.048700	-0.389600	-1.428800
30	1	0	-2.989400	-1.806600	-1.625000
31	1	0	-4.591300	-2.057500	1.485100
32	1	0	-3.240600	-1.299200	2.492500
33	1	0	1.956000	2.187200	-1.638800
34	1	0	0.521900	1.247500	-2.042000
35	1	0	0.420500	3.007400	-1.888900
36	1	0	-2.205400	2.017800	-1.916900
37	1	0	2.532600	-2.507700	-1.976700
38	1	0	2.485100	-3.343300	-0.411000
39	1	0	0.988900	-3.036900	-1.301800
40	1	0	0.951700	-0.205200	2.422900

Conformer 3b

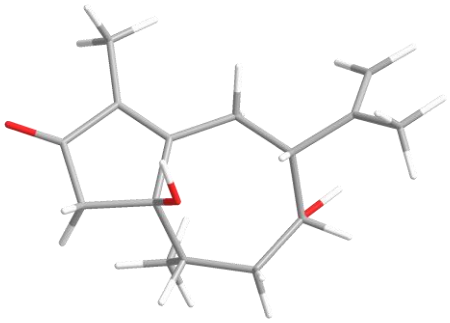

Table S21. Standard orientation of 3b.

Center Number	Atomic Number	Atomic Type	Coordinates (Angstroms) X	Y	Z
1	6	0	-1.132800	-0.769600	0.635700
2	6	0	-0.826700	0.574400	0.025800
3	6	0	0.571600	1.051100	-0.261700
4	6	0	1.759900	0.279600	0.363100
5	6	0	1.977600	-1.123600	-0.257200
6	6	0	0.980700	-2.199500	0.182800
7	6	0	-0.522600	-1.988300	-0.099200
8	6	0	-2.671300	-0.844100	0.685900
9	6	0	-3.080800	0.487900	0.145100
10	6	0	-1.921300	1.300900	-0.256700
11	8	0	-4.236400	0.871800	0.042200
12	6	0	3.077800	1.080100	0.290800

13	6	0	4.041300	0.832600	1.426100
14	6	0	3.389400	1.961100	-0.676700
15	6	0	-0.839500	-2.034500	-1.601200
16	8	0	2.008400	-1.063800	-1.683900
17	6	0	-2.093100	2.640300	-0.867600
18	8	0	-0.661200	-0.758700	1.986800
19	1	0	0.688500	1.085800	-1.352600
20	1	0	0.632200	2.090900	0.090300
21	1	0	1.553200	0.161200	1.434300
22	1	0	2.964000	-1.499200	0.044500
23	1	0	1.271900	-3.141300	-0.303500
24	1	0	1.116700	-2.373000	1.258300
25	1	0	-1.000300	-2.879500	0.336500
26	1	0	-3.094900	-1.622700	0.046400
27	1	0	-3.053200	-0.955100	1.705800
28	1	0	4.956600	1.426100	1.327500
29	1	0	3.576700	1.098700	2.381100
30	1	0	4.334300	-0.220900	1.462900
31	1	0	4.331200	2.504000	-0.665400
32	1	0	2.728400	2.174800	-1.510400
33	1	0	-1.916600	-2.107600	-1.778900
34	1	0	-0.481100	-1.149300	-2.133000
35	1	0	-0.379100	-2.913600	-2.065100
36	1	0	2.762600	-0.496000	-1.923400
37	1	0	-2.725000	2.575600	-1.759300
38	1	0	-2.567400	3.325300	-0.157800
39	1	0	-1.139100	3.080300	-1.171700
40	1	0	-0.975300	0.058500	2.412300

Conformer 3c

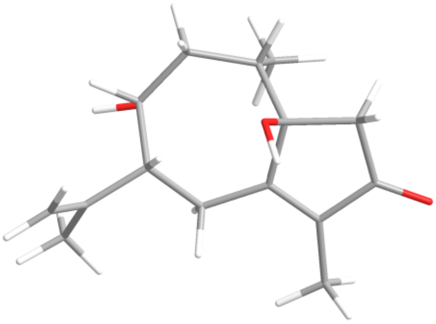

Table S22. Standard orientation of 3c.

Center Number	Atomic Number	Atomic Type	Coordinates (Angstroms) X	Y	Z
1	6	0	1.146300	0.751700	0.659100
2	6	0	0.791500	-0.582200	0.050800

3	6	0	-0.624300	-1.039500	-0.177500
4	6	0	-1.775500	-0.208900	0.431600
5	6	0	-1.960900	1.182400	-0.220000
6	6	0	-0.939600	2.235500	0.225600
7	6	0	0.558900	1.990800	-0.062500
8	6	0	2.687400	0.781800	0.680200
9	6	0	3.049900	-0.539600	0.083300
10	6	0	1.860500	-1.324100	-0.285100
11	8	0	4.193300	-0.939000	-0.080100
12	6	0	-3.093300	-1.007200	0.412900
13	6	0	-3.535100	-1.670400	-0.865300
14	6	0	-3.832400	-1.133300	1.530100
15	6	0	0.873700	2.042600	-1.565200
16	8	0	-1.954700	1.110300	-1.644400
17	6	0	1.981200	-2.660500	-0.914300
18	8	0	0.702800	0.754000	2.019300
19	1	0	-0.700900	-2.058800	0.227800
20	1	0	-0.773600	-1.127200	-1.261700
21	1	0	-1.545400	-0.043000	1.492100
22	1	0	-2.943900	1.578600	0.070500
23	1	0	-1.210800	3.185500	-0.256400
24	1	0	-1.069200	2.407700	1.302100
25	1	0	1.057700	2.867400	0.379200
26	1	0	3.120500	1.573900	0.064200
27	1	0	3.092000	0.843100	1.695700
28	1	0	-4.485900	-2.200800	-0.739700
29	1	0	-3.682700	-0.937800	-1.661300
30	1	0	-2.799800	-2.408800	-1.198000
31	1	0	-4.758800	-1.700700	1.541600
32	1	0	-3.535100	-0.673800	2.468600
33	1	0	1.950800	2.113300	-1.744000
34	1	0	0.513200	1.160000	-2.099900
35	1	0	0.416200	2.925200	-2.025300
36	1	0	-2.877400	0.995900	-1.928900
37	1	0	2.562800	-2.596100	-1.839500
38	1	0	2.485900	-3.356900	-0.237300
39	1	0	1.005900	-3.086900	-1.166500
40	1	0	0.976200	-0.085400	2.429400

Conformer 3d

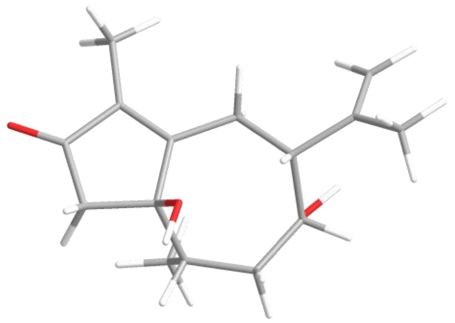

Table S23. Standard orientation of 3d.

Center Number	Atomic Number	Atomic Type	Coordinates (Angstroms) X	Y	Z
1	6	0	-1.134900	-0.771900	0.632500
2	6	0	-0.824300	0.578600	0.041400
3	6	0	0.574300	1.062600	-0.229800
4	6	0	1.763600	0.273600	0.368300
5	6	0	1.969700	-1.117700	-0.282100
6	6	0	0.972800	-2.198900	0.146000
7	6	0	-0.531800	-1.981000	-0.124800
8	6	0	-2.673400	-0.831600	0.691800
9	6	0	-3.076700	0.502400	0.153500
10	6	0	-1.914300	1.315100	-0.234300
11	8	0	-4.233400	0.878900	0.030500
12	6	0	3.085900	1.068300	0.299800
13	6	0	4.055700	0.796000	1.424000
14	6	0	3.394500	1.966700	-0.652500
15	6	0	-0.859000	-2.002700	-1.624900
16	8	0	1.987800	-1.035100	-1.707500
17	6	0	-2.078000	2.665500	-0.821600
18	8	0	-0.654600	-0.760700	1.979500
19	1	0	0.690400	1.129700	-1.319400
20	1	0	0.636400	2.091300	0.153200
21	1	0	1.566000	0.134300	1.438700
22	1	0	2.957300	-1.502300	0.003700
23	1	0	1.259500	-3.133200	-0.357200
24	1	0	1.114600	-2.390200	1.217700
25	1	0	-1.009500	-2.877700	0.299300
26	1	0	-3.105100	-1.608400	0.055400
27	1	0	-3.052900	-0.937300	1.713200
28	1	0	4.973400	1.386400	1.329700
29	1	0	3.598900	1.047400	2.386700
30	1	0	4.343500	-0.259400	1.440200
31	1	0	4.338400	2.505800	-0.636900

32	1	0	2.727000	2.201900	-1.475000
33	1	0	-1.937800	-2.069800	-1.795600
34	1	0	-0.501800	-1.110200	-2.145300
35	1	0	-0.404600	-2.875800	-2.105700
36	1	0	2.720700	-0.438400	-1.943400
37	1	0	-2.703000	2.618800	-1.719300
38	1	0	-2.556500	3.338600	-0.103200
39	1	0	-1.120800	3.109200	-1.110100
40	1	0	-0.938300	-1.588600	2.404500

Conformer 3e

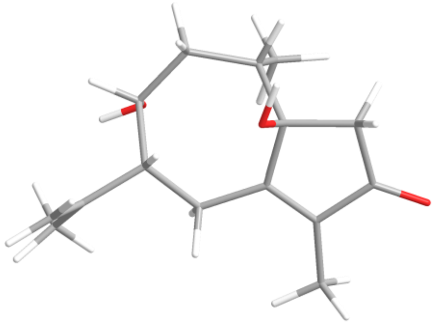

Table S24. Standard orientation of 3e.

Center Number	Atomic Number	Atomic Type	Coordinates (Angstroms) X	Y	Z
1	6	0	1.142500	0.720700	0.695200
2	6	0	0.772300	-0.598000	0.062700
3	6	0	-0.647200	-1.054400	-0.145700
4	6	0	-1.791100	-0.185700	0.420000
5	6	0	-1.929200	1.192700	-0.270000
6	6	0	-0.915500	2.236400	0.217400
7	6	0	0.590700	1.979600	-0.023200
8	6	0	2.683100	0.711400	0.744600
9	6	0	3.028800	-0.577500	0.073200
10	6	0	1.830600	-1.340500	-0.305200
11	8	0	4.169100	-0.962700	-0.141600
12	6	0	-3.116500	-0.971200	0.410900
13	6	0	-3.692500	-1.442100	-0.897000
14	6	0	-3.746100	-1.262000	1.564200
15	6	0	0.960200	2.055000	-1.512700
16	8	0	-1.837600	1.104900	-1.689900
17	6	0	1.932300	-2.666300	-0.958700
18	8	0	0.673000	0.692800	2.045100
19	1	0	-0.795700	-1.191200	-1.224700
20	1	0	-0.733800	-2.053700	0.304900

21	1	0	-1.575200	0.008300	1.478400
22	1	0	-2.921900	1.604800	-0.043100
23	1	0	-1.162300	3.190800	-0.268800
24	1	0	-1.079500	2.404600	1.289700
25	1	0	1.083000	2.843200	0.450300
26	1	0	3.143400	1.533700	0.190900
27	1	0	3.071500	0.696700	1.768200
28	1	0	-4.509100	-2.157200	-0.745700
29	1	0	-4.103900	-0.602600	-1.463100
30	1	0	-2.938900	-1.948200	-1.506200
31	1	0	-4.675200	-1.824700	1.582600
32	1	0	-3.350000	-0.951700	2.527200
33	1	0	2.043100	2.132200	-1.649600
34	1	0	0.624400	1.178600	-2.073300
35	1	0	0.517300	2.942800	-1.976800
36	1	0	-2.697500	0.801200	-2.022700
37	1	0	2.483700	-2.586200	-1.901000
38	1	0	2.459500	-3.372700	-0.309600
39	1	0	0.950000	-3.090900	-1.185000
40	1	0	0.986000	1.501200	2.486600

Conformer 3f

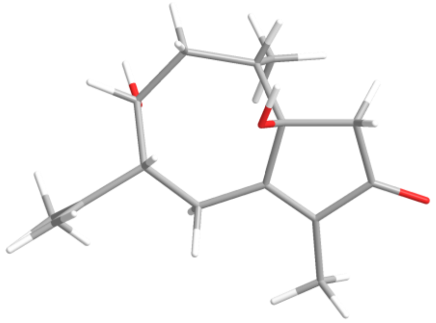

Table S25. Standard orientation of 3f.

Center Number	Atomic Number	Atomic Type	Coordinates (Angstroms) X	Y	Z
1	6	0	1.145100	0.713500	0.701900
2	6	0	0.778300	-0.599300	0.057100
3	6	0	-0.640400	-1.060200	-0.149000
4	6	0	-1.784500	-0.211500	0.444800
5	6	0	-1.940100	1.180200	-0.213500
6	6	0	-0.925500	2.224500	0.268400
7	6	0	0.577300	1.976200	0.002000
8	6	0	2.686100	0.714700	0.735900
9	6	0	3.034300	-0.565400	0.049200
10	6	0	1.837900	-1.331800	-0.327200

11	8	0	4.175500	-0.939400	-0.180200
12	6	0	-3.103500	-1.004600	0.395400
13	6	0	-3.711400	-1.308700	-0.948700
14	6	0	-3.699700	-1.426200	1.525200
15	6	0	0.920800	2.064300	-1.492700
16	8	0	-1.898000	1.092100	-1.637300
17	6	0	1.941200	-2.648900	-0.997600
18	8	0	0.687000	0.668900	2.054800
19	1	0	-0.797000	-1.177900	-1.229200
20	1	0	-0.716800	-2.069000	0.282000
21	1	0	-1.573900	-0.045300	1.509000
22	1	0	-2.935100	1.569200	0.042600
23	1	0	-1.184100	3.183700	-0.201500
24	1	0	-1.073500	2.378600	1.345200
25	1	0	1.073900	2.837800	0.474400
26	1	0	3.134400	1.545200	0.184600
27	1	0	3.085300	0.693500	1.755300
28	1	0	-4.588800	-1.959000	-0.859200
29	1	0	-4.040900	-0.390500	-1.443200
30	1	0	-2.996300	-1.823600	-1.596400
31	1	0	-4.627700	-1.990000	1.503500
32	1	0	-3.274600	-1.226100	2.504400
33	1	0	2.001600	2.135200	-1.648300
34	1	0	0.567900	1.196500	-2.056100
35	1	0	0.475300	2.959800	-1.939400
36	1	0	-2.178100	1.953900	-1.990900
37	1	0	2.486900	-2.554900	-1.941900
38	1	0	2.475000	-3.361000	-0.360400
39	1	0	0.959400	-3.074900	-1.224000
40	1	0	1.021300	1.459500	2.512000

Conformer 3g

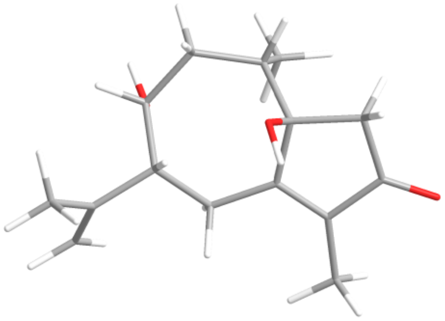

Table S26. Standard orientation of 3g.

Center	Atomic	Atomic	Coordinates (Angstroms)		

Number	Number	Type	X	Y	Z
1	6	0	1.121000	0.742200	0.668300
2	6	0	0.813300	-0.585500	0.022500
3	6	0	-0.585800	-1.060900	-0.269800
4	6	0	-1.768100	-0.277400	0.346900
5	6	0	-1.970800	1.127500	-0.270700
6	6	0	-0.984400	2.193500	0.218600
7	6	0	0.524800	1.984400	-0.041400
8	6	0	2.659600	0.805400	0.733300
9	6	0	3.067800	-0.501800	0.134600
10	6	0	1.907100	-1.302100	-0.287500
11	8	0	4.223900	-0.876800	0.006400
12	6	0	-3.085200	-1.072800	0.272400
13	6	0	-3.963100	-0.983900	1.493600
14	6	0	-3.466800	-1.802100	-0.790700
15	6	0	0.870000	2.073100	-1.535400
16	8	0	-1.948200	1.066600	-1.697600
17	6	0	2.074900	-2.623400	-0.937500
18	8	0	0.640800	0.707100	2.015500
19	1	0	-0.697800	-1.103900	-1.360900
20	1	0	-0.654100	-2.098100	0.088400
21	1	0	-1.565700	-0.158500	1.418800
22	1	0	-2.972900	1.484500	0.002400
23	1	0	-1.264700	3.148300	-0.248000
24	1	0	-1.141300	2.338700	1.295400
25	1	0	0.996400	2.861300	0.428700
26	1	0	3.092100	1.612100	0.136000
27	1	0	3.034100	0.866100	1.760300
28	1	0	-4.897400	-1.542400	1.372700
29	1	0	-3.441900	-1.392900	2.365200
30	1	0	-4.224500	0.058700	1.700600
31	1	0	-4.407000	-2.345500	-0.801400
32	1	0	-2.866500	-1.858100	-1.693600
33	1	0	1.949500	2.162800	-1.689800
34	1	0	0.533300	1.197500	-2.096500
35	1	0	0.409000	2.958800	-1.986000
36	1	0	-2.279800	1.919000	-2.028700
37	1	0	2.693500	-2.531500	-1.836000
38	1	0	2.561900	-3.325800	-0.253900
39	1	0	1.118000	-3.059000	-1.239400
40	1	0	0.909700	-0.140700	2.411600

Conformer 3h

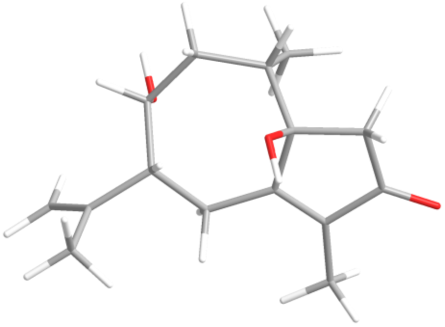

Table S27. Standard orientation of 3h.

Center Number	Atomic Number	Atomic Type	Coordinates (Angstroms) X	Y	Z
1	6	0	1.118500	0.629100	0.782500
2	6	0	0.751300	-0.599700	-0.012100
3	6	0	-0.668000	-1.024500	-0.281700
4	6	0	-1.816400	-0.199600	0.338200
5	6	0	-1.909400	1.247700	-0.210300
6	6	0	-0.907000	2.220000	0.424700
7	6	0	0.602800	1.972400	0.203600
8	6	0	2.657800	0.600900	0.864000
9	6	0	3.009900	-0.583200	0.022800
10	6	0	1.813500	-1.287400	-0.464900
11	8	0	4.150400	-0.947300	-0.222400
12	6	0	-3.128000	-0.994400	0.193300
13	6	0	-3.492600	-1.858800	1.372900
14	6	0	-3.920600	-0.956100	-0.891600
15	6	0	1.018400	2.220500	-1.255000
16	8	0	-1.780600	1.285100	-1.631100
17	6	0	1.919900	-2.517300	-1.284900
18	8	0	0.617000	0.474500	2.112200
19	1	0	-0.810700	-1.075100	-1.369800
20	1	0	-0.760000	-2.058100	0.081800
21	1	0	-1.656800	-0.114000	1.420600
22	1	0	-2.909900	1.636800	0.021800
23	1	0	-1.130400	3.226600	0.044400
24	1	0	-1.104600	2.266400	1.503600
25	1	0	1.087700	2.766400	0.792600
26	1	0	3.136700	1.489900	0.446000
27	1	0	3.020800	0.437300	1.884100
28	1	0	-4.417100	-2.421000	1.203600
29	1	0	-2.697400	-2.582700	1.577000
30	1	0	-3.638900	-1.240400	2.264500
31	1	0	-4.836500	-1.536500	-0.949900

32	1	0	-3.672100	-0.358900	-1.763500
33	1	0	2.105500	2.300000	-1.350700
34	1	0	0.691600	1.421900	-1.926000
35	1	0	0.597800	3.163000	-1.622200
36	1	0	-1.988300	2.191700	-1.916400
37	1	0	2.470600	-2.313500	-2.208800
38	1	0	2.450000	-3.299900	-0.732900
39	1	0	0.939000	-2.912800	-1.563800
40	1	0	0.844100	-0.421000	2.419100

Conformer 3i

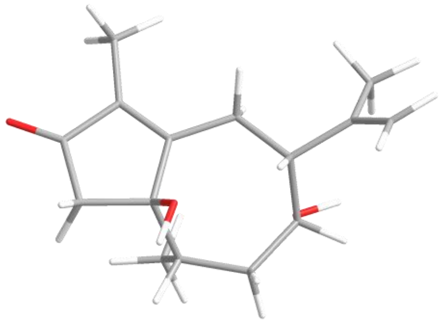

Table S28. Standard orientation of 3i.

Center	Atomic	Atomic	Coordinates (Angstroms)		
Number	Number	Type	X	Y	Z
1	6	0	-1.146700	-0.762300	0.654600
2	6	0	-0.786700	0.586600	0.086400
3	6	0	0.629100	1.056400	-0.110400
4	6	0	1.785100	0.190000	0.434700
5	6	0	1.945000	-1.172900	-0.281500
6	6	0	0.925800	-2.238100	0.140600
7	6	0	-0.575500	-1.979000	-0.117300
8	6	0	-2.687400	-0.772900	0.693700
9	6	0	-3.042900	0.553200	0.105300
10	6	0	-1.850500	1.341900	-0.237100
11	8	0	-4.186200	0.945400	-0.079000
12	6	0	3.114700	0.970800	0.423900
13	6	0	3.487500	1.782000	-0.789300
14	6	0	3.923400	0.954600	1.499200
15	6	0	-0.914100	-1.978300	-1.615600
16	8	0	1.914100	-1.046300	-1.701000
17	6	0	-1.962300	2.698000	-0.823400
18	8	0	-0.684300	-0.789800	2.007000
19	1	0	0.776600	1.217100	-1.186800
20	1	0	0.706000	2.045400	0.363800

21	1	0	1.575600	-0.019900	1.491900
22	1	0	2.930000	-1.589600	-0.029100
23	1	0	1.186500	-3.170800	-0.379400
24	1	0	1.070300	-2.449700	1.208100
25	1	0	-1.071900	-2.868200	0.301200
26	1	0	-3.134600	-1.560800	0.082200
27	1	0	-3.082700	-0.830200	1.713200
28	1	0	4.478200	2.238500	-0.684000
29	1	0	3.516200	1.163600	-1.688600
30	1	0	2.774400	2.596700	-0.947000
31	1	0	4.857200	1.509300	1.521100
32	1	0	3.673200	0.391600	2.394000
33	1	0	-1.994500	-2.037100	-1.778800
34	1	0	-0.556900	-1.080000	-2.126000
35	1	0	-0.469100	-2.847300	-2.112400
36	1	0	2.801900	-0.767100	-1.981500
37	1	0	-2.526000	2.663300	-1.761100
38	1	0	-2.481600	3.370700	-0.133500
39	1	0	-0.983600	3.134700	-1.042100
40	1	0	-0.994700	-1.618100	2.411900

Conformer 3j

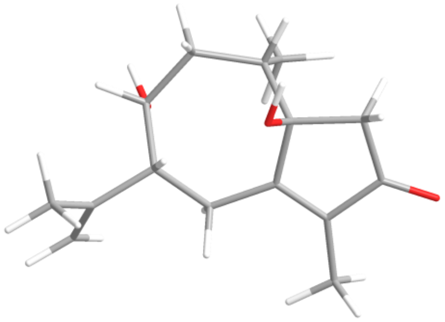

Table S29. Standard orientation of 3j.

Center Number	Atomic Number	Atomic Type	Coordinates (Angstroms) X	Y	Z
1	6	0	1.123500	0.737100	0.674700
2	6	0	0.806100	-0.592500	0.039200
3	6	0	-0.595400	-1.071900	-0.232600
4	6	0	-1.774900	-0.263600	0.353100
5	6	0	-1.958300	1.130800	-0.292500
6	6	0	-0.969700	2.197300	0.192000
7	6	0	0.540800	1.976600	-0.052400
8	6	0	2.662400	0.778400	0.748500
9	6	0	3.059300	-0.524100	0.133700
10	6	0	1.892500	-1.318400	-0.276300

11	8	0	4.214900	-0.891000	-0.023700
12	6	0	-3.096500	-1.051200	0.274400
13	6	0	-3.942700	-1.019500	1.519600
14	6	0	-3.504200	-1.727100	-0.814000
15	6	0	0.901400	2.048400	-1.543600
16	8	0	-1.916200	1.049900	-1.717700
17	6	0	2.046200	-2.646800	-0.914300
18	8	0	0.631500	0.695600	2.016500
19	1	0	-0.708700	-1.154100	-1.321400
20	1	0	-0.668700	-2.095300	0.162600
21	1	0	-1.585400	-0.123800	1.424700
22	1	0	-2.961300	1.498600	-0.036700
23	1	0	-1.240500	3.147400	-0.289300
24	1	0	-1.134800	2.357000	1.265500
25	1	0	1.013900	2.855200	0.412900
26	1	0	3.105800	1.590100	0.166100
27	1	0	3.034900	0.817500	1.777300
28	1	0	-4.881200	-1.570200	1.396000
29	1	0	-3.400800	-1.469700	2.357600
30	1	0	-4.195500	0.013100	1.781100
31	1	0	-4.444400	-2.269800	-0.829800
32	1	0	-2.920800	-1.740800	-1.729700
33	1	0	1.983000	2.130000	-1.687300
34	1	0	0.565100	1.169200	-2.099300
35	1	0	0.450400	2.932300	-2.007600
36	1	0	-2.279600	1.881600	-2.066700
37	1	0	2.649300	-2.565900	-1.824400
38	1	0	2.543900	-3.342800	-0.231700
39	1	0	1.083600	-3.084200	-1.194400
40	1	0	0.939500	1.496500	2.474500

Conformer 3k

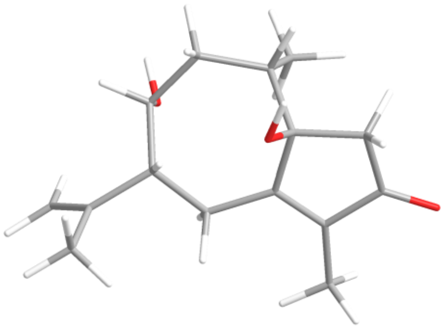

Table S30. Standard orientation of 3k.

Center	Atomic	Atomic	Coordinates (Angstroms)		

Number	Number	Type	X	Y	Z
1	6	0	1.118200	0.622900	0.793900
2	6	0	0.741000	-0.611900	0.015100
3	6	0	-0.680600	-1.045300	-0.224700
4	6	0	-1.826400	-0.180300	0.341400
5	6	0	-1.884100	1.247600	-0.260100
6	6	0	-0.883700	2.227500	0.367600
7	6	0	0.627600	1.961600	0.181500
8	6	0	2.655000	0.563700	0.892600
9	6	0	2.996700	-0.606200	0.028900
10	6	0	1.795000	-1.307600	-0.445500
11	8	0	4.135600	-0.954700	-0.246500
12	6	0	-3.148700	-0.959000	0.199500
13	6	0	-3.525600	-1.808700	1.386500
14	6	0	-3.939900	-0.920500	-0.886500
15	6	0	1.075700	2.180800	-1.272100
16	8	0	-1.719900	1.236500	-1.677200
17	6	0	1.888400	-2.548200	-1.249900
18	8	0	0.594500	0.477400	2.114800
19	1	0	-0.826300	-1.161400	-1.307600
20	1	0	-0.775900	-2.054300	0.201300
21	1	0	-1.687100	-0.057400	1.423000
22	1	0	-2.883800	1.658900	-0.065300
23	1	0	-1.091100	3.225500	-0.043200
24	1	0	-1.100300	2.304100	1.441100
25	1	0	1.109200	2.761100	0.765600
26	1	0	3.152300	1.456100	0.504100
27	1	0	3.003600	0.367300	1.912000
28	1	0	-4.458700	-2.357900	1.221900
29	1	0	-2.742100	-2.543400	1.596800
30	1	0	-3.662400	-1.181000	2.273100
31	1	0	-4.862200	-1.491200	-0.940100
32	1	0	-3.682400	-0.336700	-1.764500
33	1	0	2.165500	2.246400	-1.345800
34	1	0	0.753300	1.375200	-1.936900
35	1	0	0.673800	3.121800	-1.663300
36	1	0	-1.933800	2.127900	-2.002000
37	1	0	2.413300	-2.353900	-2.190700
38	1	0	2.438400	-3.319300	-0.701100
39	1	0	0.903500	-2.955000	-1.496600
40	1	0	0.935100	1.211200	2.654900
